# Territoriality and the organization of technology during the Last Glacial Maximum in southwestern Europe

**DOI:** 10.1371/journal.pone.0225828

**Published:** 2019-12-11

**Authors:** João Cascalheira

**Affiliations:** ICArEHB, University of Algarve, Faro, Portugal; Max Planck Institute for the Science of Human History, GERMANY

## Abstract

Climate changes that occurred during the Last Glacial Maximum (LGM) had significant consequences in human eco-dynamics across Europe. Among the most striking impacts are the demographic contraction of modern humans into southern refugia and the potential formation of a population bottleneck. In Iberia and southern France transformations also included the occurrence of significant technological changes, mostly marked by the emergence of a diverse set of bifacially-shaped stone projectiles. The rapid dissemination of bifacial technologies and the geographical circumscription of specific projectile morphologies within these regions have been regarded as evidence for: (1) the existence of a system of long-distance exchange and social alliance networks; (2) the organization of human groups into cultural facies with well-defined stylistic territorial boundaries. However, the degree and modes in which cultural transmission have occurred within these territories, and how it may have influenced other domains of the adaptive systems, remains largely unknown. Using southern Iberia as a case-study, this paper presents the first quantitative approach to the organization of lithic technology and its relationship to hunter-gatherers’ territorial organization during the LGM. Similarities and dissimilarities in the presence of morphological and metric data describing lithic technologies are used as a proxy to explore modes and degrees of cultural transmission. Statistical results show that similarities in technological options are dependent on the chronology and geographical distance between sites and corroborate previous arguments for the organization of LGM settlement in Southern Iberia into discrete eco-cultural facies.

## Introduction

Centered around c. 21 ka cal BP, the Last Glacial Maximum (LGM) was a global climate event characterized by the maximum expansion of the ice sheet over Scandinavia and northern Europe, along with generally cold and arid conditions in central and western Europe [1–3].

Climatic changes occurred during this period had substantial repercussions in the geography, technology, and social behavior of human populations in Europe. Among the most drastic impacts are: (1) the contraction of the human range to southern regions of Europe that served as refugia, such as the Iberian and the Italian peninsulas [4–11]; (2) the consequent genesis of a bottleneck scenario in human genetic diversity [12–14]; (3) and, in southwestern Europe, the advent of a set of new technologies for the manufacture of lithic projectiles and knives using bifacial retouch.

This latter feature is one of the main elements traditionally used to define the so-called Solutrean techno-complex [15]. The Solutrean is a phenomenon geographically limited to southwestern France and the Iberian Peninsula, presenting a moderately short chronological range (c. 25–19 ka cal BP) when compared to other pan-European techno-complexes and representing, to a certain extent, an outlier within the common background persisting throughout the Upper Paleolithic in western Europe.

For its uniqueness and rough coincidence with the LGM, the Solutrean record often emerges as a promising context for examining the mechanisms of formal and chronological change of lithic tools during the late Pleistocene. Further, it has been recognized as an excellent opportunity to test the possible links between the development of specific technical innovations and social behavior under a particular climate setting [16–18].

In fact, besides the long-standing recognition of the several types of bifacial implements as secure chrono-cultural markers, their presence across southwestern Europe, and the notion that they may convey complex symbolic communication, has inspired many theories and models on social networks, territorial organization, demographic changes, and the subsistence of hunter-gatherers.

Two perspectives have been particularly relevant over time. One that denotes the existence of a system of long-distance exchange and alliance networks, based on the identification of uniform techno-economic patterns across all of southern France and Iberia (including the use of bifacial technologies, but also the presence of similar patterns in raw material selection, systematic use of heat treatment and stylistic similarities in art) [19–21]. The other, in contrast, based on the recognition of spatially-patterned bifacial typological traditions (e.g., Cantabrian vs Mediterranean) [15,22–24], and justified by the existence of environmental/ecological constraints [7,25], seem to have resulted in the delineation of stylistic territorial boundaries and an inherent recognition of social group ownership by populations inhabiting rather distant regions [26,27].

Overall, these patterns attest a complex form of sharing behaviors occurring across different scales during the LGM. From a demographic point of view, this implies the existence of a very dynamic population circulating over significantly large territories, resulting in the permutation of different kinds of information, including, very likely, genetic exchange [20,28].

Since most models on LGM territoriality, however, are based on the study of bifacial and shouldered projectiles, there is a significant lack of information on how cultural transmission, on the one hand, and regional ecology, on the other, may have influenced other domains of the adaptive systems. Notably, it is still largely unknown to what extent technological organization and, mostly, mental templates for lithic reduction sequences, were shaped by territorial organization during this period. Are similarities within regions only expressed at the symbolic/stylistic level, with communities sharing projectile morphologies and artistic elements to express belonging to a common territory? Or, on the other hand, did the contexts in which long-distance contacts seem to be present also exhibit similarities at other levels of the technological system, including, for example, central tendencies in the lithic reduction sequences?

Answering these questions becomes particularly relevant given that bifacial tools, albeit important, are just a singular component in LGM technological structures. Solutrean industries are mostly composed of a non-bifacial techno-typological substrate [29,30], comparable to that present at any other Upper Paleolithic techno-complex [31], that formed the basis for the production of a wide range of blanks and tools.

In this context, this paper provides the first quantitative comparison between the non-bifacial components of LGM lithic assemblages from a set of sites located at geographically distant regions but within the same stylistic/cultural territory. Using southern Iberia as a case-study, specific technological domains of the assemblages are statistically compared to explore scenarios of territoriality and the organization of technology across the LGM.

## LGM archaeology in southern Iberia

**Fig 1:**
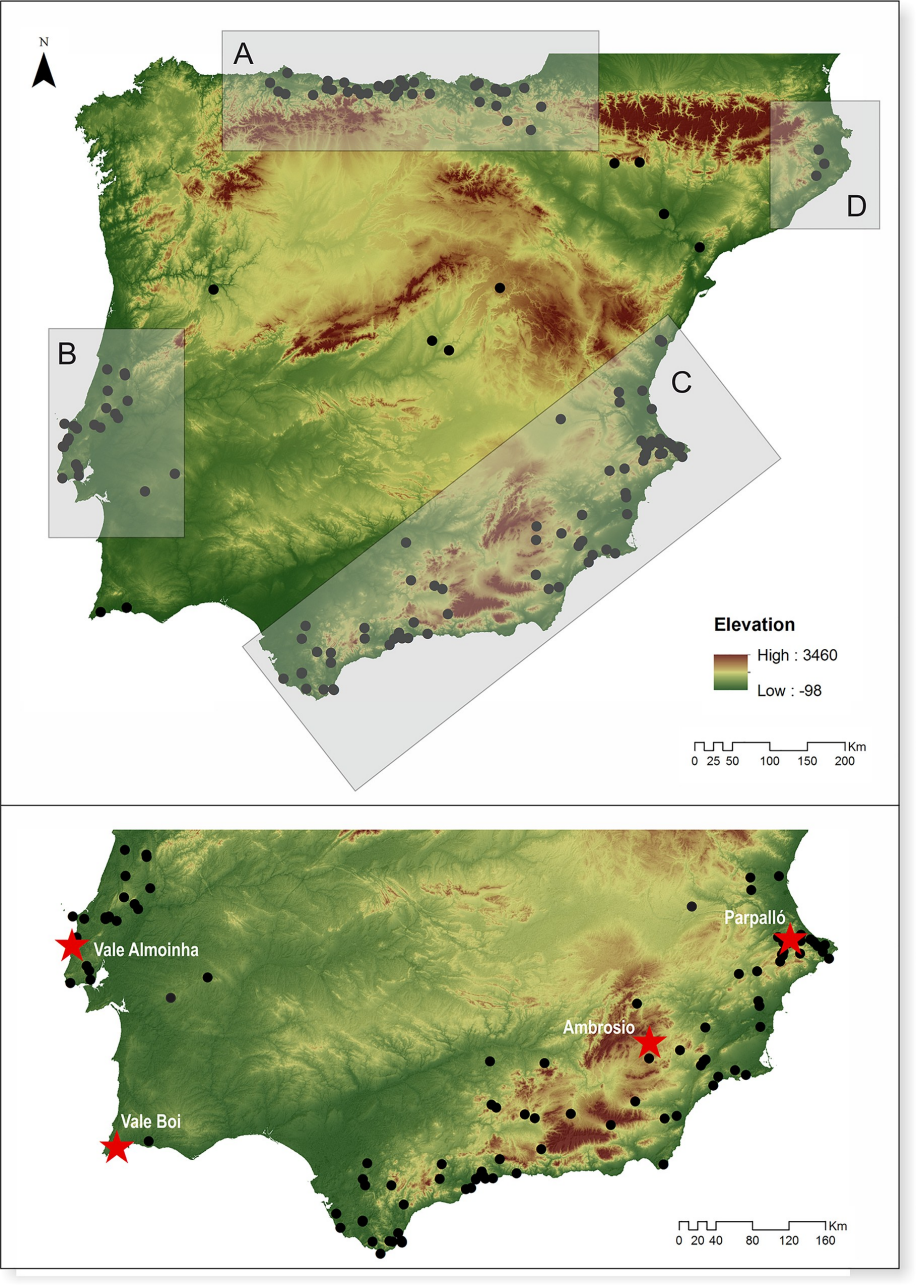
**Top—Geographical distribution of the cultural facies (A- Cantabrian, B- Portuguese, C- Mediterranean, D-Catalan) defined for the LGM in Iberia. Bottom—location of LGM sites in the southern part of the Peninsula, sites used in this study are marked with a star.** Topographic data from the U.S. Geological, Survey Shuttle Radar Topography Mission (https://earthexplorer.usgs.gov/).

In Iberia, four cultural facies are distinguished based on the distribution of bifacial and/or shouldered point types during the LGM (Fig 1). The southern part of the Peninsula encompasses two of them, known as Mediterranean and Portuguese facies [23]. Both have in common the presence of abruptly retouched shouldered points [32] and stemmed and winged projectiles [33,34]. They are, however, separated by the fact that at some sites from central Portugal Cantabrian-type shouldered points (with bifacial and flat retouch) are also documented [24]. This separation between central Portugal and the remaining territory also became evident in previous analysis of stemmed and winged projectiles morpho-metrics. Points from central Portugal are generally larger, in both length and width [26,35], and present significantly different stem shapes [36] than the ones from southern Portugal and Mediterranean Spain. While these patterns seem to indicate some distinction between regions, the exclusive distribution of both projectile morphologies below 40° N latitude conventionally attests the validity of using southern Iberia as a broad cultural/stylistic unit. Artistic particularities, mostly from mobile art, also corroborate this territorial homogeneity [37–41].

In chronological terms, according to the traditional models [21,23,24,42–44], backed shouldered projectiles and stemmed and winged points are only characteristic of the later phases of the Solutrean techno-complex (i.e., Upper Solutrean and Solutreo-Gravettian). However, this interpretation has been questioned in a recent Bayesian analysis of the currently available radiocarbon data [45], calling into doubt the status of those type-fossils as precise temporal markers, and confirming their presence throughout most of the LGM time-span (i.e., c. 25–19 ka cal BP).

LGM settlement of southern Iberia is marked by the existence of more or less dense clusters of sites, that are relatively isolated from one another by geographical hiatus where the complete absence or the presence of very few sites might testify human passage but not likely intensive occupation [46,47]. Caves and rock-shelters are better represented than open-air locations, as are multi-component sites over single horizon ones [27,45]. There is evidence for the existence of different types of sites, including long-term and seasonal residential camps, shorter specialized occupations, lithic cache sites, and possible aggregation sites [48]. The number of occupied sites is significantly higher than during the early Upper Paleolithic and, although they tend to be in coastal or near-coastal settings, inland camps are also represented, mainly as single-occupation localities [49,50].

Rather problematic is the quality of the available data from most of these sites. More than half of the occurrences are surface or mixed contexts, suffering from several limitations (e.g., lack of organic materials, presence of small and/or highly truncated lithic assemblages, and occurrence of thick palimpsest deposits—[21,27,45,51,52]).

Probably as a result of this scenario, technological analyses of LGM lithic materials are (as well as faunal analysis) very limited in southern Iberia. These are mostly restricted to intra-site descriptions and evaluation of the assemblages [23,35,46,47,53] and, very rarely, presenting inter-site or regional comparisons using technological patterns for the construction of clear paleoanthropological testable hypothesis [54].

Two rare examples are the studies conducted by Zilhão [24] and Schmidt [36]. The first, focusing on central Portugal, uses assemblage composition and raw material circulation to report a scenario of logistically organized resource-procurement strategies within the region. Body adornments distribution and technological data allowed the author to further suggest the organization of the region into smaller territories corresponding to different ethnic groups, sharing specific traditions but all inserted within the same ethnic boundary.

The second study addresses Iberia as a whole and compares the technological organization behind the manufacture and use of the different types of lithic projectiles. Previously referred significant differences between northern and southern Iberia are corroborated in this study, and the main outcome is that synchronic variability of Solutrean points between those regions does not only relate to stylistic variations, but to regional differences deeply rooted in the distinct technological strategies of tool making and tool use. Corroborating Zilhão [24] the author states that: “the technological design of southern Iberian points (…) encompasses criteria of toolkits ascribed to logistical mobility of collectors, with a high functional specialization of the points, and evidence at `base camps’ for batch production” [36].

Both models seem to confirm the scarce data coming from faunal studies [55,56] that demonstrate a seasonal movement of residential sites between coastal areas and the mountainous hinterland, and possibly a perennial occupation of few major sites across southern Iberia [54].

In sum, paleoanthropological models for LGM hunter-gatherer adaptations in southern Iberia are currently rather limited [20,57,58] and mostly based on the typo-technological analysis of bifacial and shouldered projectiles or other “style-embedded” elements. Some of these elements report, on the one hand, significantly homogeneous patterns across the region and, on the other, regional particularities that suggest the organization of settlement into smaller socio-cultural territories.

## Theoretical framework for cultural transmission processes

The diffusion of specific concepts across southern Iberia, as rapidly as it seems to have occurred during the LGM, may have resulted from an array of very diverse contact behaviors. Ethnoarchaeological data [59–61] has demonstrated that the process of social interaction between hunter-gatherer communities “can be understood to vary between events in which individuals have little exposure to each others’ residential space, with meetings occurring at the edge of their respective ranges, to events in which marriage partners are exchanged and previously ‘foreign’ individuals become fixtures in each others’ residential lives” [62,63]. Contacts occurring anywhere between these two extremes of the contact scale result in dissimilar access to information, and to different types of information.

In the case of LGM projectiles and remaining shared concepts, both hypotheses are viable to have occurred. The design concepts of shouldered and stemmed and winged projectile points may have been disseminated in intimate cultural environments with access to the full production sequences, or it may, on the contrary, be the outcome of independent innovation by exposure to curated tools and not necessarily to the complete technological mechanisms. Within this latter scenario, although it is possible that an individual could independently develop some of the behaviors of point production from examining a finished tool, it is rather unlikely that he/she would use the same procedures, techniques, and methods, during the knapping process. Thus, understanding the complete production process is of great importance to distinguish between modes but mostly degrees of social contact, and how these vary across time. Focusing exclusively on information coming from projectiles morphologies, as well as on any other class of traditionally considered style-embedded items (i.e., portable art, ornaments, lithic retouched tools), will only allow to explore the patterns of presence/absence of contacts and not the possible nature of those contacts. Further, using LGM bifacial reduction sequences and projectile manufacture in southern Iberia to shed light on the technological processes is a somehow hampered task. Patterns of segregation of the production sequence, which comprise shaping of preforms at sites close to rich raw material sources on the one hand, and export of preforms and finished points to other locations on the other hand [23,36], can severely truncate the variability of technological procedures. Since, however, the available information on point production attests, in a large number of cases, that bifacial points were obtained from blanks produced within non-bifacial reduction sequences [24,35,64], a reasonable approach is to look at the non-bifacial technology of the assemblages and check for patterns of similarity/dissimilarity in the distribution of specific methods and concepts within the reduction sequence continuum.

Such kind of analysis requires, however, a theoretical and methodological framework that would allow the integration of both social interactions and ecological background as shapers of stone knapping patterns across space and time. While there are many ethnoarchaeological and theory-based studies addressing the meaning of morphological variations of lithic tools as indicators of style and territorial differentiation, very few attempts have been made to use technological processes as proxies for cultural transmission processes. The middle-range theory presented by Tostevin [62,63], for studying the material evidence of social contact between Neanderthals and Anatomically Modern Humans using lithic technology, is perhaps one of the most compelling and sophisticated approaches. Based on a body of literature on artifact style [59,65–69], optimization studies [70–73] and experimental knapping [74–77], Tostevin’s model seeks to identify whether variation in lithic material culture style reflects one or more social processes, once other sources of variability have been dismissed.

The present paper adopts the same body of theory to inform a technology-based approach (see methods below).

Ethnography has shown that technological gestures are not just individual action sequences with clearly defined goals, but suites of distributed behaviors reflecting complex systems that have been learned and assimilated as bodily techniques. In this context, flintknapping processes are considered “technological performances which can only be effectively taught and learned by observation of the body movements, the handling of core and percussor, and the flake by flake strategizing on how to exploit core volumes” [63]. Lithic reduction is a directional and irreversible process characterized by a sequence of mechanical relationships. While these relationships are influenced by multiple contextual, aspects, such as the quality of the available raw material, identifying different exploitation patterns within these mechanical relationships are thought to reflect a purposeful choice of particular modes of technological manipulation present in an assemblage out of an equifinal pool of alternative possibilities—what Sackett [68,78,79] have called isochrestic variation.

Based on this theoretical approach, it is assumed here that the characterization and comparison of the central tendencies of lithic attributes distribution within specific and comparable clusters of technological decisions can be used as indicators of behavior differentiation. In this way, it is possible to assess how knappers responsible for different assemblages solve problems, react to different circumstances and produce artifacts according to specific learned methods, resulting in the variability detected among assemblages. It is also assumed thus that technological and technical choices, whether conscious or not, taken by knappers are integral components of cultural life and are “largely dictated by the technological traditions within which they have been enculturated as members of the social groups that delineate their ethnicity” [68]. The same choices can though be subject to revision as a result of changes in the patterns of social interaction between groups.

Consequently, the greater the similarities recorded between assemblages, the greater the probability of concepts exchange to have occurred between knappers responsible for different archaeological assemblages, most likely in a context of social intimacy, with full access to technological conceptual schemes. On the contrary, if dissimilarities are more evident, than either technological practices transmission occurred in the context of a low degree of social intimacy, and/or ecological and cultural [80,81] regional constraints did not allow the generalization of basic technological patterns.

This study builds, therefore, upon the theoretical assumption that the amount of lithic technological knowledge, learned and adopted by hunter-gatherers, depends upon how and where learning occurs and may reflect differential access to particular enculturating environments, and thus “are determined by the mechanics of how the traits are transmitted from individual to individual and by the stochastic events associated with these” [82].

The model expectations for different modes of cultural transmission during the LGM in southern Iberia based on technological analysis of lithic attributes presented in this paper are summarized in Table 1. Similar approaches using lithic technology as a proxy for demography and cultural transmission have been applied in several other studies [62,83–85].

**Table 1:** Model expectations for different degrees of cultural transmission during the LGM in southern Iberia, based on technological analysis of lithic attributes.

Hypothesis	Expectations	Caveats
LGM contacts occurred with high social intimacy, at residential spaces, with possible long-term movement of people. Contact with the complete technological process	Assemblages recurrently show no statistical significant differences under a range of different measures	Equifinality and restricted options within the technological processes
LGM contacts occurred in pathways or temporary aggregation sites with short-term movement of people. Contact with curated tools	Assemblages recurrently show statistical significant differences under a range of different measures	Functional organization; Ecological limitations (e.g. raw material constraints); Chronological differences

Some caveats are associated with the expectations presented since they result from a complex interplay among patterns of inheritance, interaction, and local adaptation. Whereas equifinality and the restricted options inherent to leptolithic technologies may result in similar technological trends, even within communities with none or very little contact; aspects such as chronology, functional variability, and ecological constraints (e.g., raw material variability) may influence the presence of significantly different lithic attributes among populations with a high degree of interaction. Most of these alternative explanations are related to several adaptive behaviors as they are organized across different temporal and geographical scales, within which technological organization is composed of a set of problem-solving processes that are responsive to conditions created by the interplay between hunter-gatherers and their environment [86]. Nonetheless, even in the presence of rather different assemblages due to specificities related with contextual aspects there is, based on the theoretical framework presented above, and based on the assumption that all behavior may, in one sense or another, be regarded as purposeful, always a bound between technological practices and the definition of a particular social/cultural unit. Contextual constraints were, however, not downplayed in the course of the analysis and interpretation of results obtained in this study. Additionally, the focus on a small-scale analysis, taking full advantage of statistical methods, and targeting meaningful comparability between contexts were purposefully used in this approach to reduce the negative impact of possible errors and uncertainty.

## Sites and materials

Sites included in this study were selected to ensure, as much as possible, chronological correspondence, spatial variability, and the integrity and comparability of contexts covering the whole region under study. The set of selected sites comprises one open-air context from central Portugal (Vale Almoinha), one rock shelter from southern Portugal (Vale Boi), and two sites—one rock shelter and one cave (respectively, Ambrosio and Parpalló)—from Mediterranean Spain Fig 1 and Table 2.

**Table 2:** Sampled sites, sectors and litho-stratigraphic contexts used in this study.

Site	Site type	Sector	Context	Classic phase attribution
Vale Almoinha (VALM)	Open-air			Middle Solutrean
Vale Boi (VB)	Rockshelter		Layer A	Upper Solutrean
			Layer B	Upper Solutrean
			Layer C	Upper Solutrean
Ambrosio (AMB)	Rockshelter	1986 campaign	Layer II	Solutreo-Gravettian
			Layer IV	Upper Solutrean
			Layer VI	Middle Solutrean
Parpalló (PAP)	Cave	Sector L	4’00–4’75	Solutreo-Gravettian
			4’75–5’25	Upper Solutrean
			5’25–6’25	Middle Solutrean

Vale Almoinha (VALM) is a single LGM occupation open-air site located in the Portuguese Estremadura. It was first excavated by Manuel Heleno in the late 1940s and relocated and dated by Zilhão in 1986 [87]. The site is located in a small inland valley at c. 1.5 km from the present-day coastline. Two radiocarbon dates on charcoal samples are available, dating the archaeological horizon to c. 24–24.5 ka cal BP [24]. Previous analysis of the lithic materials [24,88] revealed an assemblage dominated by chert, some quartzite and residual presence of quartz. Core maintenance products are well represented. Lithic analysis revealed the presence of two distinct approaches, one for the specific production of laurel-leaves and another to produce flakes and elongated blanks. Laurel-leaf points are the well-most represented tool type, followed by unifacial flat-retouched points, willow-leafs and two, questionable [36], shouldered points. The overall characteristics of the assemblage and site context allowed Zilhão [24] to characterize it as a semi-permanent base camp, in which are represented the various phases of the *chaîne operatoire*.

Vale Boi (VB) is a multilayered and multicomponent (open-air and rock shelter) site located in the southwesternmost extreme of Iberia (southern Portugal). The site is situated in the eastern side of a wide valley, 2.5 km inland from the present-day coastline, occupying a wide portion of a relatively steep slope, marked by the presence of a 10–15 m high limestone cliff on the top. LGM occupations are attested across all excavated areas, but the most complete sequence was identified below the boulders resulting from the collapse of a relatively large rock shelter [89]. Six radiocarbon dates indicate that Solutrean occupations at VB rock shelter started somewhere around 25 ka cal BP and ended shortly before c. 20.5 ka cal BP [45,47]. Previous analysis of lithic assemblages revealed the presence of backed shouldered and stemmed and winged points across the sequence of three lithostratigraphic units defined during excavation. Chert, quartz, and greywacke are the most common lithic materials recovered, all provisioned locally, from deposits situated no more than 15–20 km from the site. The density and diversity of findings, together with data from faunal analysis [55,90], allowed the classification of VB as a seasonal residential camp.

Ambrosio (AMB) rock shelter is located in the eastern part of Andaluzia, positioned at an elevation of 1060 meters above sea level, and distant 60 km from the Mediterranean coast. The shelter has a south-southwest orientation and a triangular shape whose entry measures c. 39 meters wide by c. 19 meters in height and extends c. 17 meters into the Miocene limestone [53]. The site was excavated by Eduardo Ripoll Perelló between 1958 and 1964 and by Sergio Ripoll Lopez from 1982 onwards, and it is mostly known for the Solutrean parietal art [91]. The Pleistocene sequence is marked by a series of Solutrean occupations organized by the excavators into three distinct horizons, separated by clast-supported levels with fewer materials. Recent AMS radiometric results contradict previous standard dates, indicating that the most recent occupation occurred at c. 24 ka cal BP. Backed shouldered and stemmed and winged points are present throughout levels II and IV but completely absent from level VI. As in the VB case, this site has been classified as a seasonal residential camp.

The Parpalló (PAP) cave is located in a wide valley, some 6 km from the present coastline. At c. 600 meters above sea level the cavity entrance is marked by a relatively narrow breach, which gives access to the main room with c. 80 m2. The first work was by Breuil in 1913. Later, between 1920 and 1930, L. Pericot undertakes a series of campaigns resulting in the almost complete excavation of the archaeological deposits [34]. With close to 8.5 meters of thickness, the stratigraphy at PAP comprises Gravettian, Solutrean and Magdalenian occupations. Unfortunately, excavation was done by subdividing the deposits into artificial spits of 20–25 centimeters and screening all sediments using large meshes [23]. Standard radiocarbon ages are available for levels 5’00–4’75 and 4’25–4’00, that, after Bayesian analysis date the horizons to c. 22.5–23 ka cal BP [45]. Shouldered and stemmed and winged points are present throughout the sequence but clearly are better represented in levels between 5.25 and 4.25 meters in depth [23,43]. This site was possibly used as a residential space, although the remarkable and unique amount of recovered engraved slabs [40] might indicate the presence of some symbolic or ritualistic functionality.

Given the size of the Spanish assemblages, materials were sampled in the case of AMB from the 1986 campaign [53], and in the case of PAP from sector L [23]. Although randomly selected, the representativeness of these samples within each site is thought to be good, given the large portion of the excavation area they represent.

For the three karstic contexts, a diachronic subdivision of the assemblages was also implemented based on the available lithostratigraphic information and previous analysis of the materials [23,43,47,53]. This subdivision resulted in a total of 10 contexts, representing, as much as possible, the chronological and geographical variability of LGM adaptations in southern Iberia.

All sites are radiocarbon dated, yet direct comparisons between contexts are problematic given the sometimes very large standard deviations of the results.

Further, all four chosen sites are located near raw material sources, and thus all assemblages have been characterized as primary flaking assemblages, containing features such as a reasonable number and diversity of core maintenance pieces and large cortical flakes. A significant difference between sites is in the diversity of raw materials used. Although chert is always dominant, in Portuguese assemblages a greater diversity of materials is present. In the case of VB, non-siliceous raw materials were used not as alternatives to chert but for very specific functional purposes [47]. This pattern is consistent with that recorded for nearly all upper Paleolithic sites in the western Atlantic facade of Iberia even when good-quality chert is available within walking distance [92]. To circumvent a likely impact on assemblage comparability caused by the use of different raw materials, only chert artifacts were selected for this study. This restriction was also imposed by the composition of the assemblages since for both Spanish sites it was not possible to identify more than a handful of artifacts made on other materials than chert, a fact that is also evident in previous publications for Ambrosio [53].

## Methods

Complete cores and blanks were analyzed using a set of technological variables and attributes, specifically chosen to allow the isolation of separate sources of variability, including raw material constraints and learned behaviors (see Supplementary Online Material [SOM] S1–S3 Tables for a complete list of attributes and measurement techniques).

Following the theoretical background of Tostevin [62,63], Scerri [93] and Scerri et al. [84], in this study variables were organized into comparable heuristic domains (i.e. Platform Maintenance, Core Exploitation, Dorsal Convexities Management, Elongated blanks Production, Core Morphology, and Core Use) of the technological sequences, to achieve a comprehensive structure of similarity and dissimilarity between assemblages. The main goal of using these domains was to isolate different sources of variability by focusing on specific sets of interrelated variables that correspond to interdependent knapping actions. Breaking down the reduction sequences into comparable clusters makes possible to account for possible biased samples and “broken” sequences, not assuming that the knapping process was restricted to a specific timeframe or the work of a single individual [84].

The chosen variables, and respective combinations, were based on the traditional parameters used in lithics attribute analysis [94,95] and the results of experimental studies available in the literature [74–77,96–100]. For example, controlled knapping experiments show that blank dimensions and shape are significantly affected by variations in exterior platform angles, platform depths, and platform preparation (e.g., faceting) [98,101]. Thus, by considering variables such as platform type and blank elongation (length/width ratio) as a set of interrelated variables, the way knappers differently exploited these variables can be assessed and compared across assemblages. The same is true for other domains, such as the exploitation of dorsal surface convexities or the management of core morphology, all with direct impact on blank morphometrics.

A multivariate statistical approach was used to explore the interaction of variables within each domain. Since most of the attributes available were categorical, a Multiple Correspondence Analysis (MCA) was used to explore the orthogonal dimensions of variability in the data. MCA is well suited to lithic analysis because it allows the isolation of the different sources of variability influencing the character of each assemblage under study. When used, metric variables were pooled into categories using a K-means clustering algorithm. To reduce the amount of ‘noise’ in the overall variability, all categorical attributes whose representation was less than 5% within each variable were also lumped together into a category named ‘Other’, before any of the analysis were performed.

Following Scerri et al. [84] the scores and loadings of the most relevant dimensions calculated by the MCA were used to identify and explore the relative contributions of aspects such as reduction intensity, raw material constraints, or particular knapping styles. To analyze the main sources of variability behind the technological character of each assemblage separately, and to assess the similarities and differences between contexts, the individual scores of each relevant component of the MCA were statistically tested by applying an analysis of variance (ANOVA). ANOVA tests and corresponding post hoc comparisons were run using a percentile t bootstrapped approach for trimmed means (trim level was set at 20%) as implemented by Mair and Wilcox [102]. This approach intended to prevent problems related to heavy-tailed distributions, unequal sample sizes, and possible differences in skewness distributions of the samples. The location of individuals, grouped by assemblages in the multivariate dimensions, was bootstrapped 5000 times in all tests.

All analyses and data processing were accomplished in R (version 3.6.1) [103]. The complete R code used for all the analysis and visualizations contained in this paper is available at http://www.doi.org/10.17605/OSF.IO/YD2VE. To produce those files the procedures described by Marwick et al. [104] for the creation of research compendiums to enhance the reproducibility of research were followed. The files provided contain all the raw data used in our analysis as well as a custom R package [105] holding the code to produce all tables and figures. To enable maximum re-use, code is released under the MIT license, data as CC-0, and figures as CC-BY (for more information see [106]).

## Results

### General characteristics of the assemblages

The total number of artifacts for each context used in this study is presented in Table 3. The most numerous assemblages are, respectively, PAP 4’00–4’75 (n = 2743) and VB B (n = 1723), and the less numerous are AMB II (n = 540), and VB A (n = 493). Cores represent between two and eight percent of the assemblages. VALM is the site with the highest percentage of cores (c. 8.52%), and a rather low blank to core ratio (10.74) when compared to the other contexts.

**Table 3:** General counts and ratios for each assemblage.

Context	Cores	Blanks	Flakes %	Elongated products %	Cortical / Non-cortical blanks ratio	Blanks / Cores ratio	Total artifacts
AMB II	10	530	44.53	55.47	0.50	53.00	540
AMB IV	14	528	65.91	34.09	0.54	37.71	542
AMB VI	13	403	70.72	29.28	0.66	31.00	416
PAP 4’00–4’75	141	2602	39.62	60.38	0.53	18.45	2743
PAP 4’75–5’25	11	652	74.08	25.92	0.79	59.27	663
PAP 5’25–6’25	15	873	86.14	13.86	1.06	58.20	888
VALM	57	612	71.24	28.76	0.61	10.74	669
VB A	20	473	80.34	19.66	0.63	23.65	493
VB B	47	1676	82.76	17.24	0.84	35.66	1723
VB C	32	830	82.77	17.23	0.70	25.94	862

Differences in a possible segregation of the reduction sequences seem not significant between sites, with small variation occurring in the ratios of cortical to non-cortical blanks. The only exceptions are the two oldest horizons from PAP and VB, which reveal the highest presence of cortical elements among all assemblages. This pattern might be related to the use of different raw material sources, or particular technological schemes applied during core reduction. The second option seems, however, the most reasonable since differences are also evident between these two contexts and the remaining assemblages, regarding the proportion of flakes and blanks with lengths equal or greater than twice their width (elongated blanks). This type of blanks is, in fact, less frequent within all complete blanks in these contexts. Additionally, a progressive change into the production of elongated products is clear at both multi-layered Spanish sites, but it is not present at the VB sequence. This chronological trend seems to go along with a process of microlitization of the elongated products during the LGM, already noted in previous works [46], and also manifested at VB [35].

**Fig 2:**
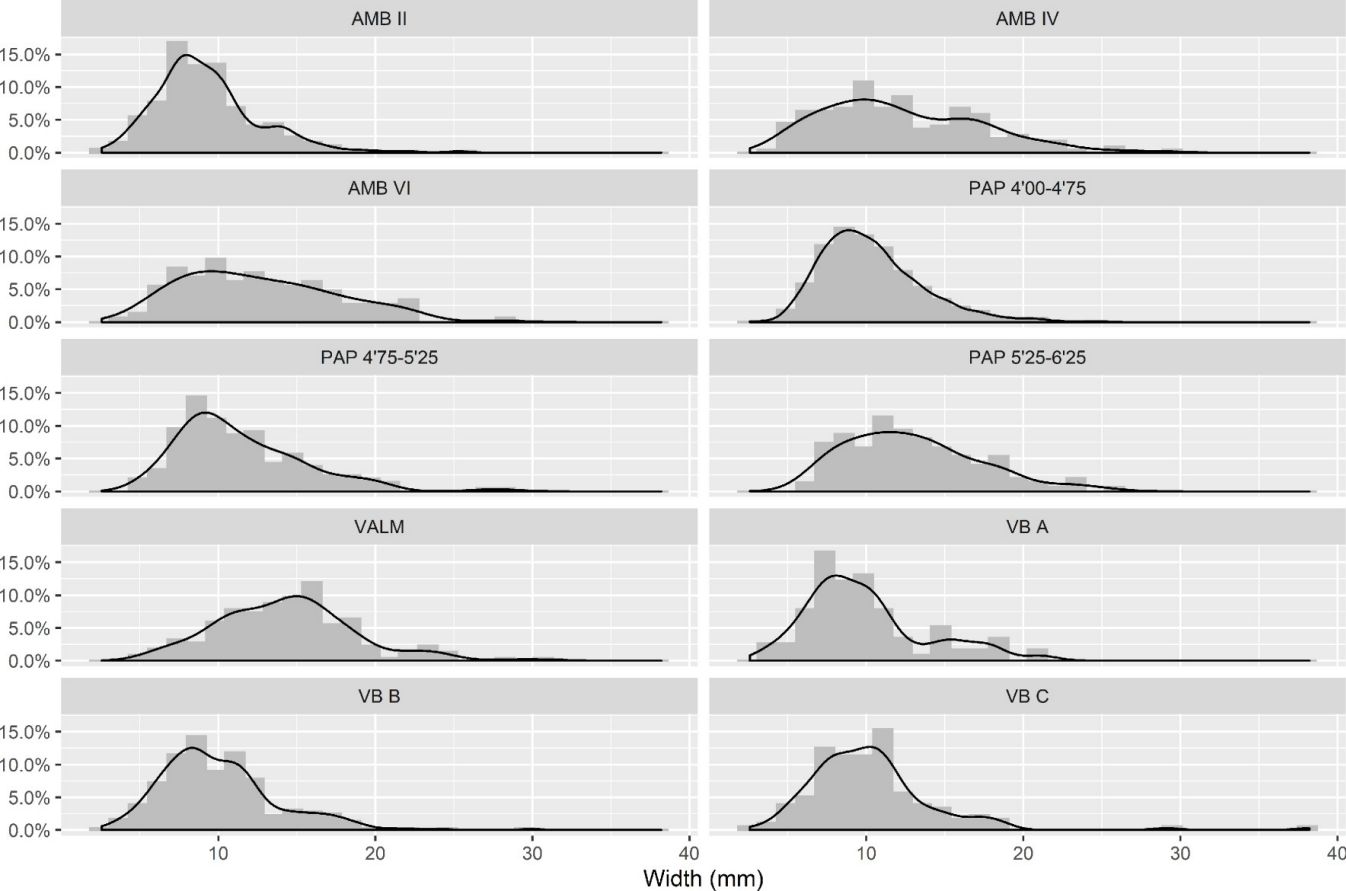
Width histograms for the elongated products from each context included in this study.

As it is shown in Fig 2, however, the reduction sequences for elongated blanks seem to have followed an unimodal tendency in all assemblages. No clear separation between blade and bladelet production is attested within contexts and thus, as in other studies [107], the traditional subdivision [108] was not used in the statistical tests below, where both classes were grouped as a single elongated blanks category.

**Fig 3:**
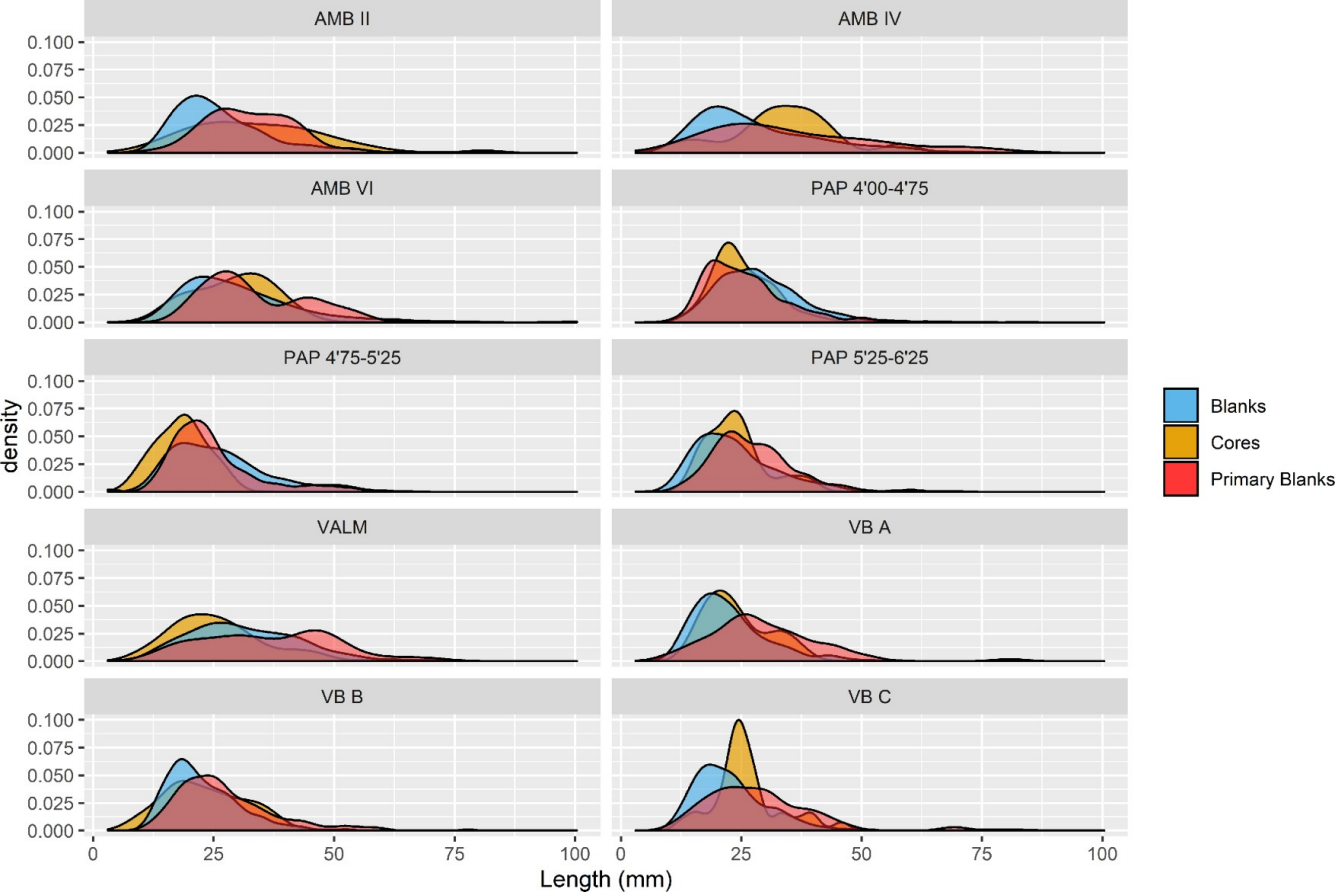
Density plots comparing the lengths of dominant blank scars on cores, blanks and fully cortical (primary) blanks. Following Henry [109] the plots show the relative flaking intensity for each assemblage.

In what regards to relative flaking intensities, there are some relevant differences among the assemblages used in this study. Fig 3 shows the comparison of density plots for three variables—maximum length of core’s dominant scar, maximum length of primary elements (considered here as blanks with more than 25% cortex on their dorsal surface), and maximum length of non-cortical blanks. Following Henry [109], an assemblage which displays core scar dimensions that are near identical to those of primary elements and fall within the upper range of the blank dimensions is one in which cores were still usable. Most of the assemblages seem to be broadly comparable in this regard, except for PAP 4’75–5’25 and VALM, which reveal a significant number of very reduced cores, presenting average scar lengths much smaller than the length of primary blanks and, more importantly, falling within the lower range of the non-cortical blanks.

**Fig 4:**
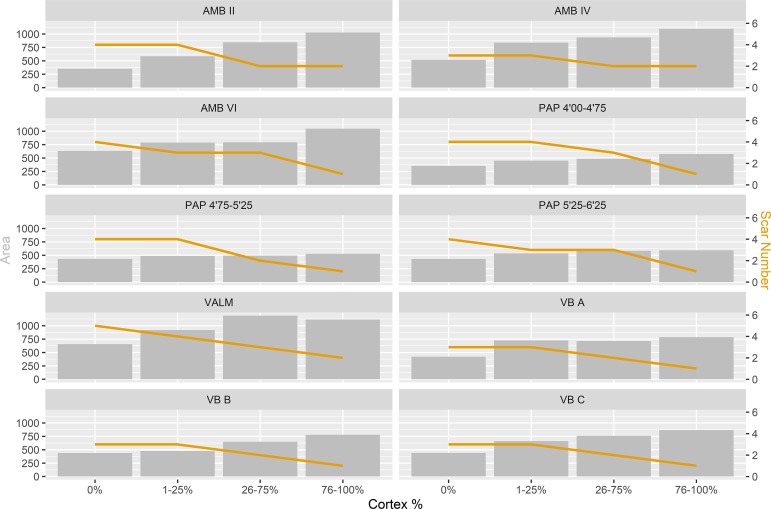
Comparison of the interaction between scar number and area of blanks by cortex frequency for each assemblage in this study.

Flaking intensity comparison is also demonstrated by the complementary analysis presented in Fig 4, which represents the interaction between blanks area (Width X Length), number of scars, and the amount of cortical surface on the dorsal surface. All assemblages present a typical decrease in the average size of blanks and percentage of cortex with an increase in the number of scars, confirming that all contexts are comparable in what concerns to the general reduction continuum. These results also imply that all three variables can be used as a good indicator for reduction intensity within each assemblage [109–112].

All these patterns seem to corroborate an overall broad comparability of the assemblages. As expected, some reservations should be taken regarding the site of VALM, which seems, in the first instance, to present technological idiosyncrasies. These are mostly related with a lower blank to core ratio, the fact that the cores seem to be more reduced than expected, and the dimensions of the elongated blanks being amongst the largest of all the studied assemblages. Two reasons may be behind this pattern: either some of the late production blanks were exported from the site, or the methods used during the excavation of the site (in the late 1940s, the early 1950s) did not allow the recovery of smaller materials. This latter hypothesis is perhaps the most parsimonious, given the small amount of chips available from the site [24].

Despite the particularities mentioned above, the overall assemblage compositions are identical. Together with the fact that all assemblages are considered primary flaking sites located close to raw materials sources, and that all have been previously classified as residential contexts, similarities in flaking intensities and reduction tendencies somehow guarantee a higher degree of certainty in the comparisons made by the tests presented below.

### Blank analysis

The analysis of blanks was organized into four domains. The first, second and third approaches considered all blanks to analyze and compare strategies of platform maintenance and the exploitation of dorsal convexity systems during the reduction sequence of cores. The fourth domain included only one class of blanks (elongated blanks), aiming to characterize and compare the organization of relevant attributes for the production of blanks with length equal or twice their width. Frequencies for all attributes recorded for blanks in each assemblage are presented as Supplementary Online Materials (S3 Table).

#### Platform maintenance

**Fig 5:**
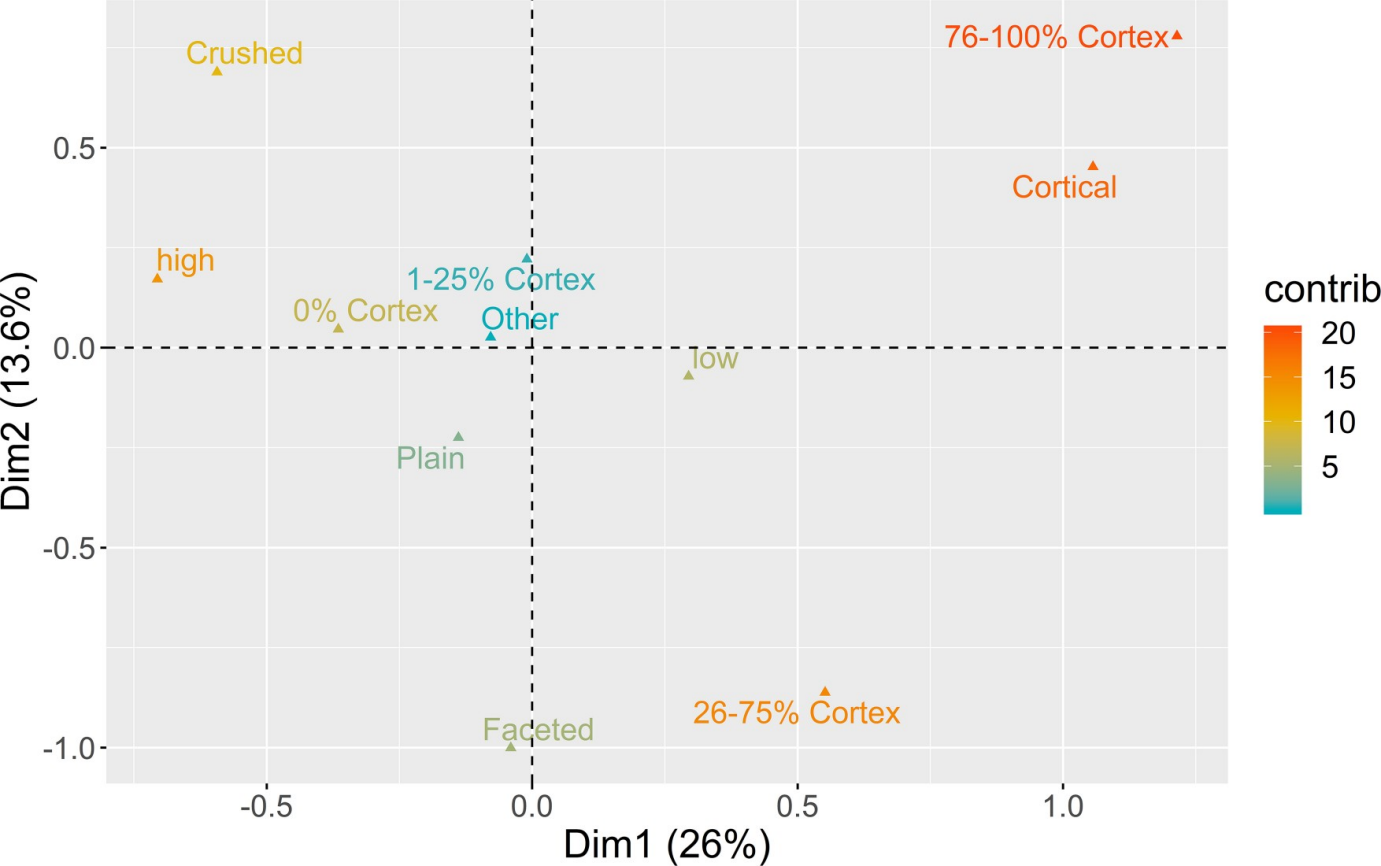
Multiple correspondence analysis plot (first two dimensions) for platform maintenance. The color ramp represents the contribution (in %) of the variable categories to the definition of the dimensions.

The analysis of platform maintenance aimed to explore how knappers managed platform characteristics to control blank dimensions and morphology across the reduction sequences. This approach was accomplished by investigating the relationship between platform type, dorsal cortex percentage, and blank elongation (length/width ratio). As in Scerri [84], the external platform angle measurements were not available for this analysis. Considering, however, the high degree of correlation between that variable and blank elongation [113], the latter was used as a proxy for possible changes in the management of the external platform angle. The results from the application of a MCA to this set of variables are presented in Fig 5.

The first two dimensions of the MCA solution represent 39.6% of the overall variability. The first dimension (26%) is marked by the correlation between blanks with cortical platforms, higher presence of cortex in the dorsal surface, and low elongation ratios, on the one hand, and blanks without dorsal cortex, crushed platforms, and high elongation, on the other hand. In the second dimension (13.6%), both cortical and crushed platforms are positively associated with high amounts of cortex, while faceted platforms are associated with medium values (26–75%) of dorsal cortex. These results suggest that for almost 40% of the overall variability in this domain, different strategies of platform preparation are associated with different stages of the reduction sequences.

**Fig 6:**
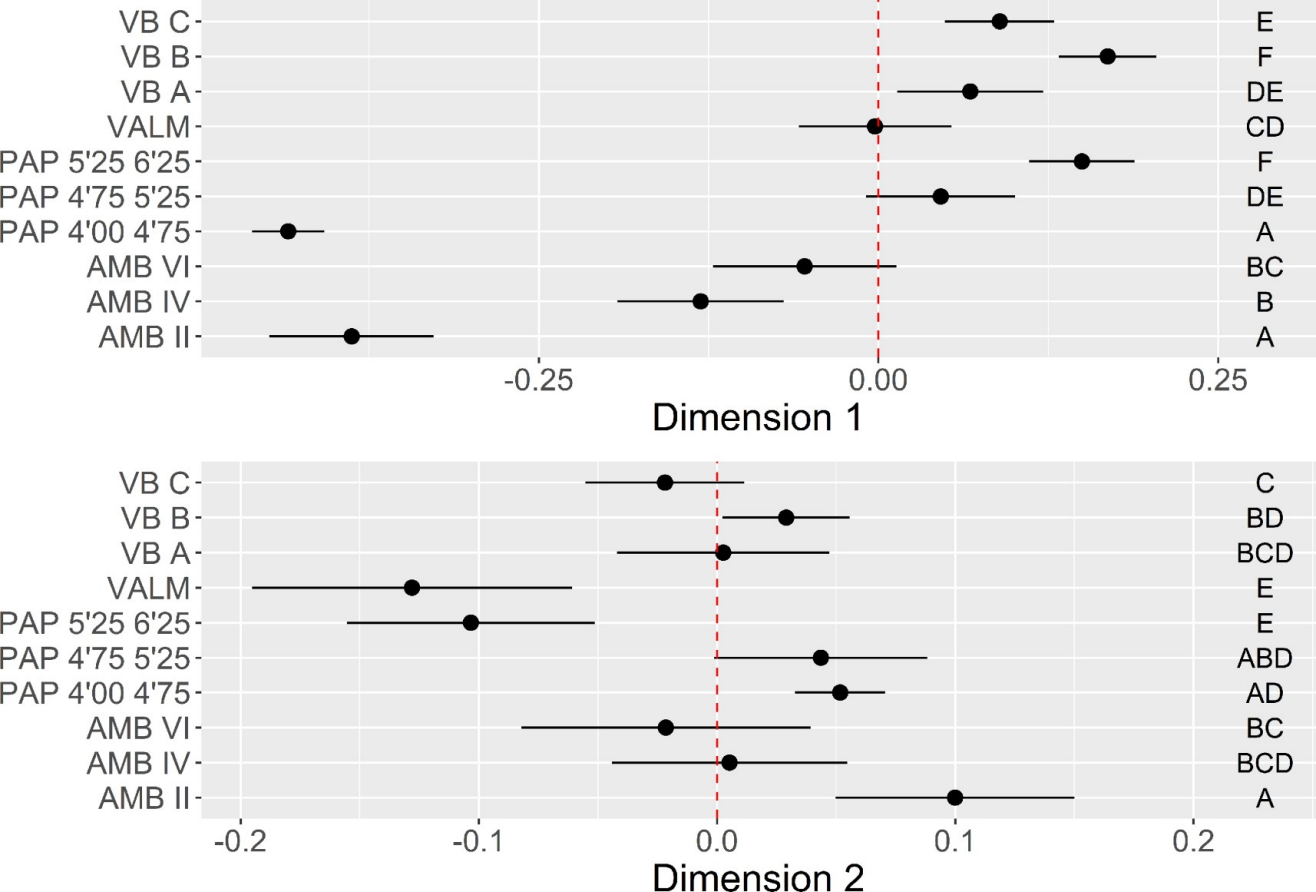
Platform maintenance multi-comparison bootstrap ANOVA. Error bars represent 95% confidence interval from the mean. Contexts sharing the same set of letters do not present statistically significant difference (p-value ≥ 0.05).

The bootstrapped ANOVA multiple comparison results for both dimensions of the MCA are shown in Fig 6. Negative loadings for Dimension 1, marked by the dominance of crushed platforms and high elongation ratios, are associated with all the assemblages from AMB and the more recent horizon of PAP, with no statistical difference detected between AMB II and PAP 4’00–4’75, on the one hand, and AMB IV and AMB VI, on the other. The remaining contexts, except for VALM, are all associated with the positive loadings of Dimension 1, marked by lower elongation ratios, and higher frequencies of dorsal cortex and cortical platforms. What seems relevant, however, is that this latter trend is counterpointed with an inversion of results in Dimension 2, with the earliest assemblages of all multi-layered sites and VALM being associated with higher frequencies of faceted platforms for the exploitation of partially decorticated flaking surfaces, not correlated with any particular elongation category. This pattern is noteworthy since it seems to show a tendency towards the abandon of platform faceting over time in multi-layered sites, more pronounced at PAP and AMB than in VB.

#### Direction of core exploitation

**Fig 7:**
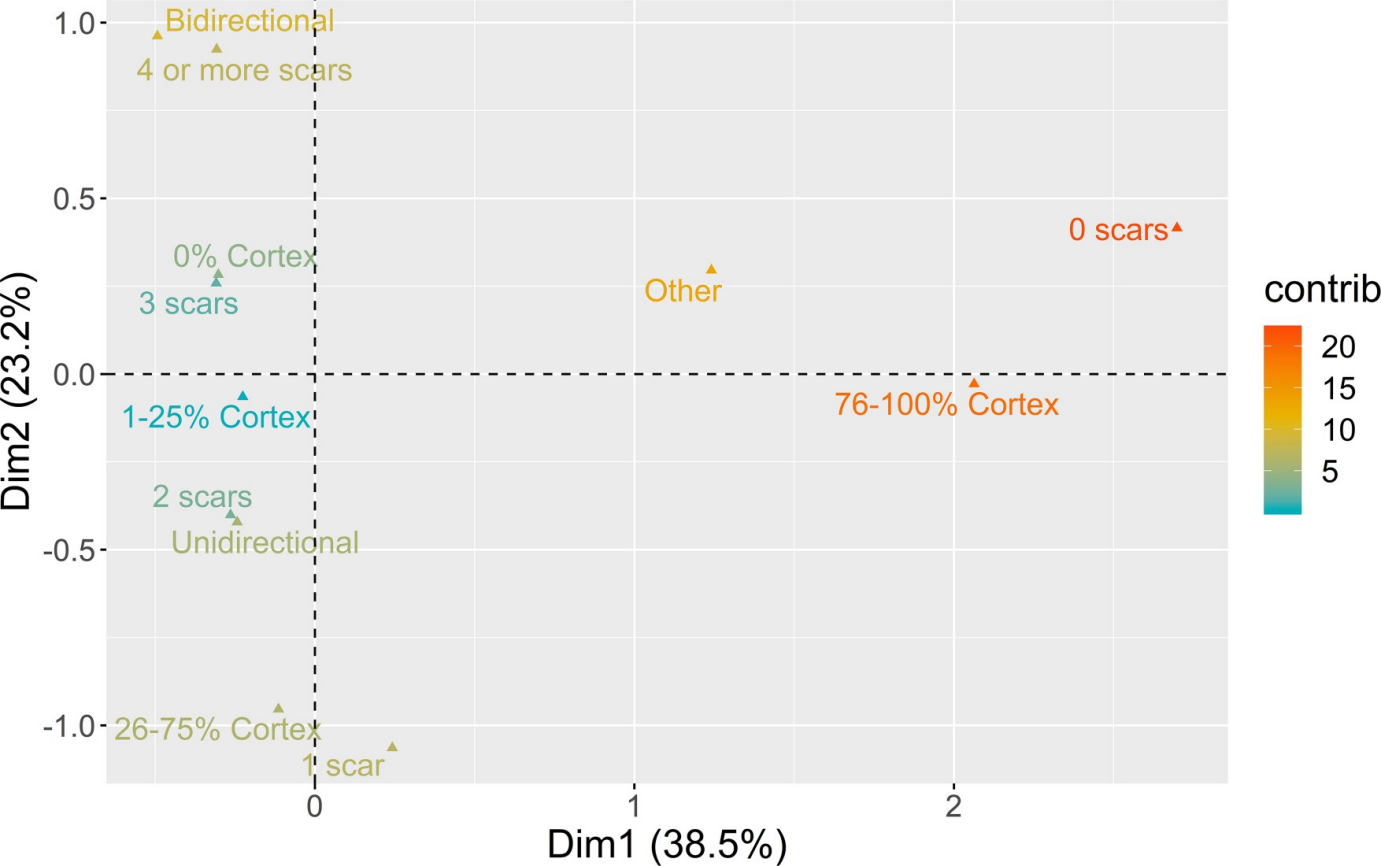
Multiple correspondence analysis plot (first two dimensions) for direction of core exploitation. The color ramp represents the contribution (in %) of the variable categories to the definition of the dimensions.

The analysis of core exploitation using blanks aimed to characterize the way cores were rotated by knappers to create different dorsal surface convexity patterns during the sequences of reduction. A combination of dorsal cortex frequency, dorsal pattern, and number of scars in the dorsal surface was used in the MCA. Results are presented in Fig 7.

The first two dimensions of the MCA solution represent close to 61% of the overall variability. Dimension 1 (38.5%) contrasts the positive loadings for other patterns of a diverse set of flaking strategies during early stages of core exploitation, to the use of bidirectional or unidirectional strategies in more advanced stages of the sequence. Dimension 2, representing 23.2%, opposes the production of blanks with few dorsal scars, using unidirectional strategies in partially decorticated flaking surfaces, to the use of bidirectional strategies from completely decorticated flaking surfaces, resulting in blanks with a high number of dorsal scars.

**Fig 8:**
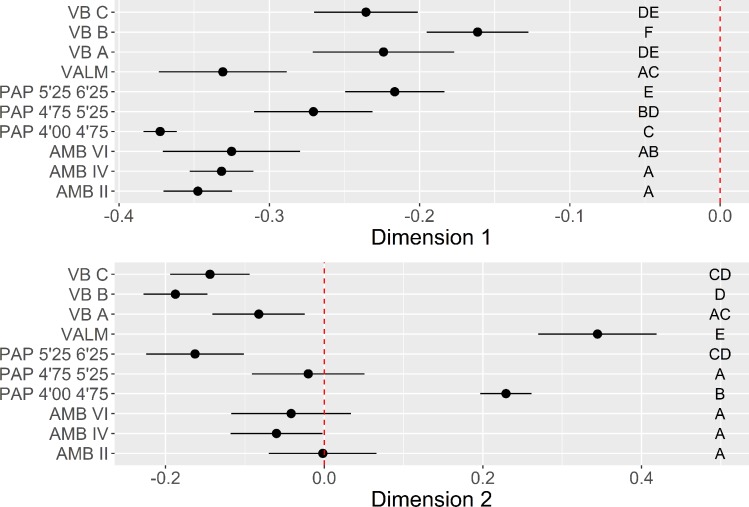
Direction of core exploitation multi-comparison bootstrap ANOVA. Error bars represent 95% confidence interval from the mean. Contexts sharing the same set of letters do not present statistically significant difference (p-value ≥ 0.05).

Fig 8 presents the results of the multi-comparison bootstrapped ANOVA to the individuals’ loadings of the MCA for the dimensions just described. While all the assemblages are only related to the negative loadings of Dimension 1. This does not imply that the initial stages of reduction are absent from the assemblages, but rather, as it would be expected, that they are, overall, less represented than later phases of the sequence. The distribution of contexts through Dimension 2 is perhaps more interest in this regard, since it shows a clear separation between VALM and PAP 4’00–4’75 (due to a dominant presence of bidirectional methods) and the remaining set of contexts. In the latter group, however, which is clearly associated with the use of unidirectional strategies on partially decorticated flaking surfaces, VB and the oldest occupation at PAP occupy the most extreme positions, being significantly different from the other PAP and AMB assemblages.

#### Dorsal surface convexity

The influence of flaking surface convexities in the overall morpho-metrics of a blank has been demonstrated by sereval authors [63,113,114]. In short, the ridge pattern of the dorsal surface of flakes determines the path of the resulting flake because the ridges guide the force of the percussor blow. Changes in the shape of the debitage surface morphology affect the dimensions of the resulting blanks by changing the distribution of mass over the said blank. In this analytical domain, a set of variables related to shape, including Elongation and Flattening ratios, Cross-section, Edge shape, and Profile are examined for all blanks. The aim of this approach is to assess patterns of blank morphological variation of each context under study, to evaluate possible differences/similiarities on how knappers controlled surface convexities during core exploitation.

**Fig 9:**
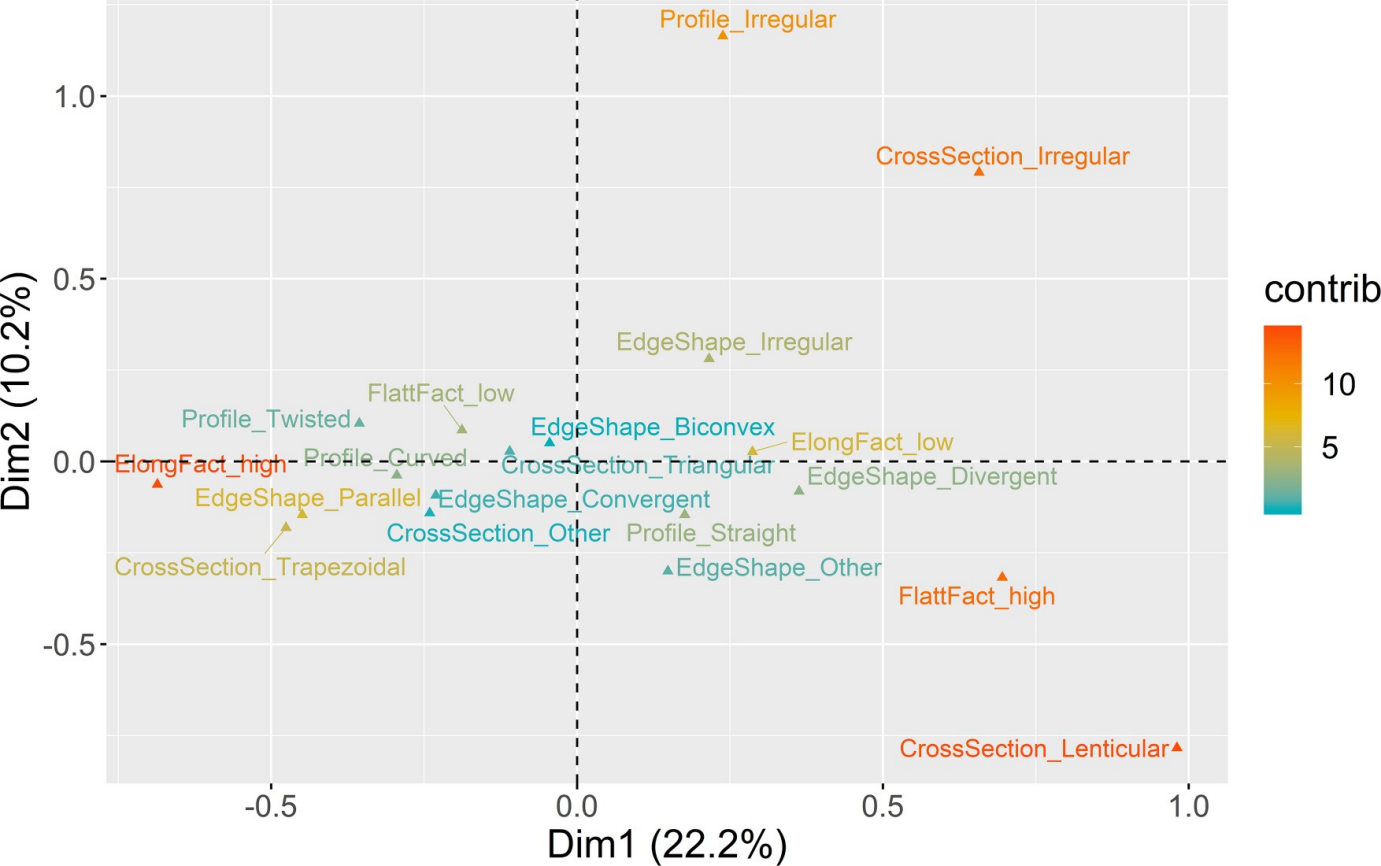
Multiple correspondence analysis plot (first two dimensions) for Dorsal surface convexity. The color ramp represents the contribution (in %) of the variable categories to the definition of the dimensions.

Fig 9 present the results of the MCA applied to these set of variables. Close to 32% of the overall variability is explained by the first two dimensions. Dimension 1 (22.2%) opposes blanks with high flattening and low elongation ratios and irregular or lenticular cross-sections, to highly elongated blanks with parallel edges and trapezoidal cross-sections. Dimension 2, on the other hand, is marked by significant positive loadings for irregular edge shapes, profiles and cross-sections, and negative loadings for high flattening ratios and lenticular cross-sections.

**Fig 10:**
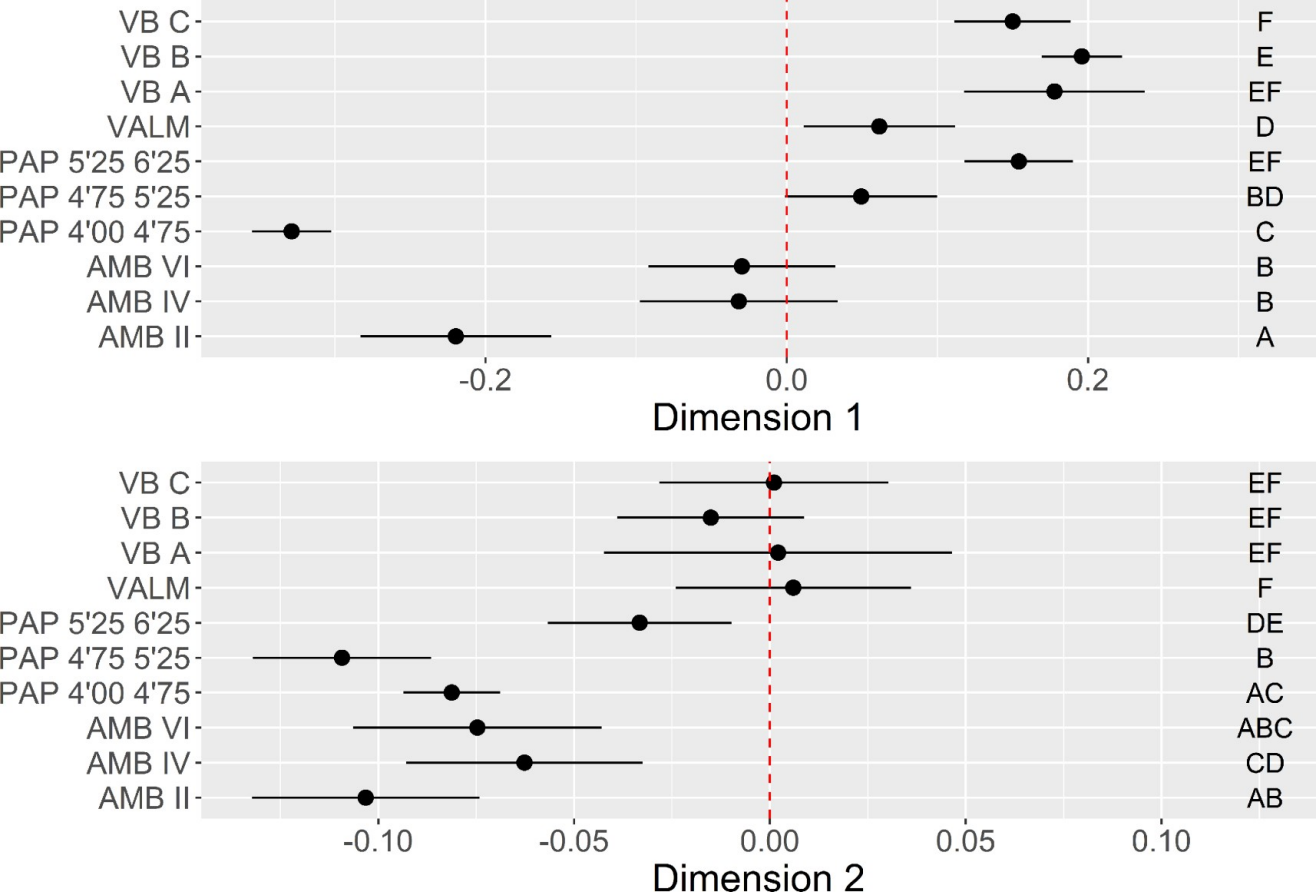
Dorsal surface convexity multi-comparison bootstrap ANOVA. Error bars represent 95% confidence interval from the mean. Contexts sharing the same set of letters do not present statistically significant difference (p-value ≥ 0.05).

The distribution of mean values from each context across the two first dimensions are presented in Fig 10. As in the previsous domains, the two youngest assemblages from PAP and AMB are clearly separated from the remaining ones, marked mostly by the predominance of high elongation ratios with parallel edges and trapezoidal cross-sections.

#### Elongated blanks techno-morphology

**Fig 11:**
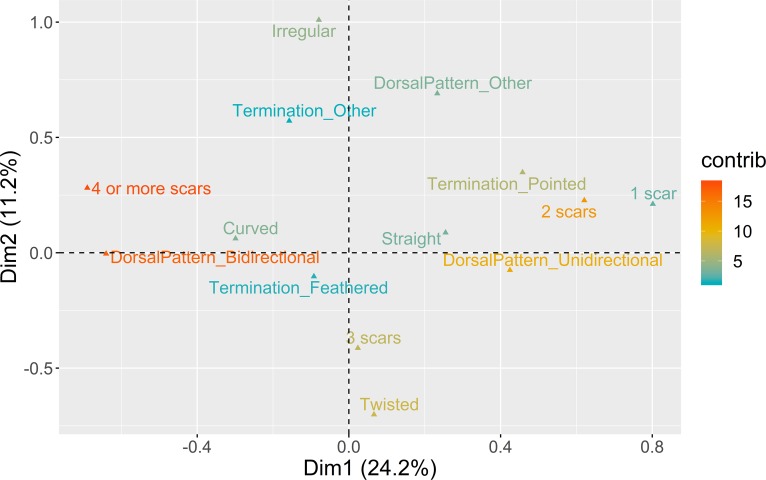
Multiple correspondence analysis plot (first two dimensions) for elongated blanks techno-morphology. The color ramp represents the contribution (in %) of the variable categories to the definition of the dimensions.

For the fourth MCA applied to the blanks sample, only the elongated blanks category was used. This isolation aimed to explore the organization of attributes in this particular technological class, given its relative importance in the tool kits of the assemblages under study. Selected variables included Dorsal pattern, Distal termination, Dorsal scar number, and Profile type. The results are presented in Fig 11.

**Fig 12:**
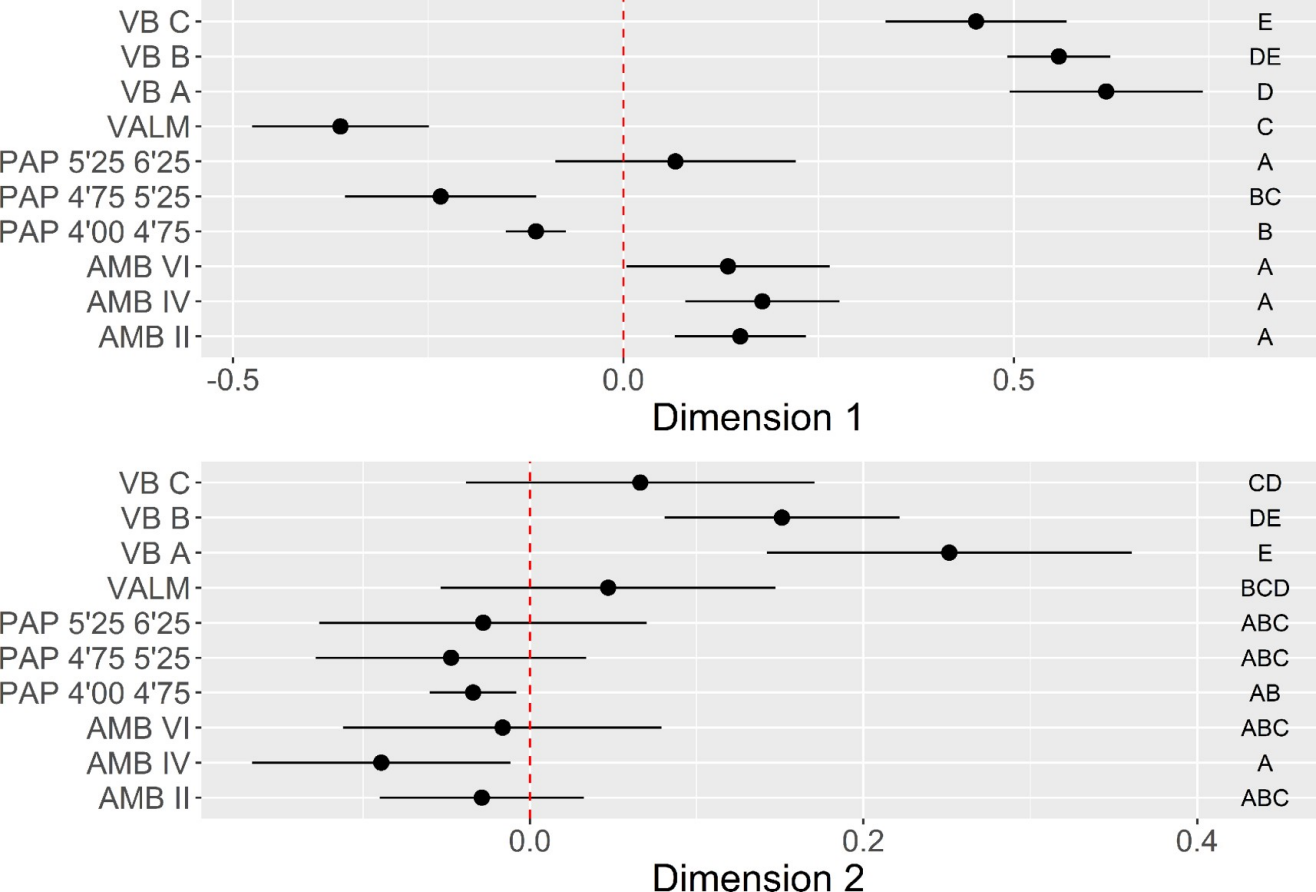
Elongated products multi-comparison bootstrap ANOVA. Error bars represent 95% confidence interval from the mean. Contexts sharing the same set of letters do not present statistically significant difference (p-value ≥ 0.05).

The first two dimensions cumulatively represent c. 37% of the overall variability. In Dimension 1 a marked distinction is made between unidirectional strategies for the production of pointed blanks with straight profiles, and bidirectional patterns used to produce a variety of distal terminations in blanks with complex dorsal biographies and curved profiles. All assemblages from VB and AMB, as well as the oldest context of PAP, are positively correlated with the positive loadings of Dimension 1 (Fig 12). However, the multi-comparison post hoc analysis denotes a significant difference between VB and the Spanish sites, underscoring the very low frequency of bidirectional strategies at VB. VALM, PAP 4’00–4’75, and PAP 4’75–5’25, on the contrary, are all driven by the negative scores of Dimension 1. In this component, VALM occupies an extreme position, mostly due to the high number of removals recorded in the dorsal surfaces of the blanks, which somehow confirms the abovementioned patterns of more intensive core reduction at this site. In Dimension 2 (11.2%), the occurrence of twisted blades and bladelets do not seem correlated with any of the other variables used. Twisted profiles are, however, better represented in all the assemblages but not at VB and VALM, which are instead dominated by a mixture of different morphologies.

### Core analysis

The analysis of cores involved two separate domains. One related to the morphological patterns of the abandoned cores, and the other to core use and variation in reduction techniques. The following points describe the overall results for the two core domains comparisons using the MCA and ANOVA multi-comparison results. The statistical results presented for cores should be carefully considered given the available sample sizes and the fact that core analysis only considers a static moment of the reduction continuum.

#### Core morphology

This domain of analysis aimed to compare central tendencies in core morpho-metrics using both metric (Elongation, Flattening, and Weight) and the categorical variables Cross-section and Core Type. The latter variable was included in this analysis since the types used in this study were mostly defined by morphological criteria (e.g., pyramidal).

**Fig 13:**
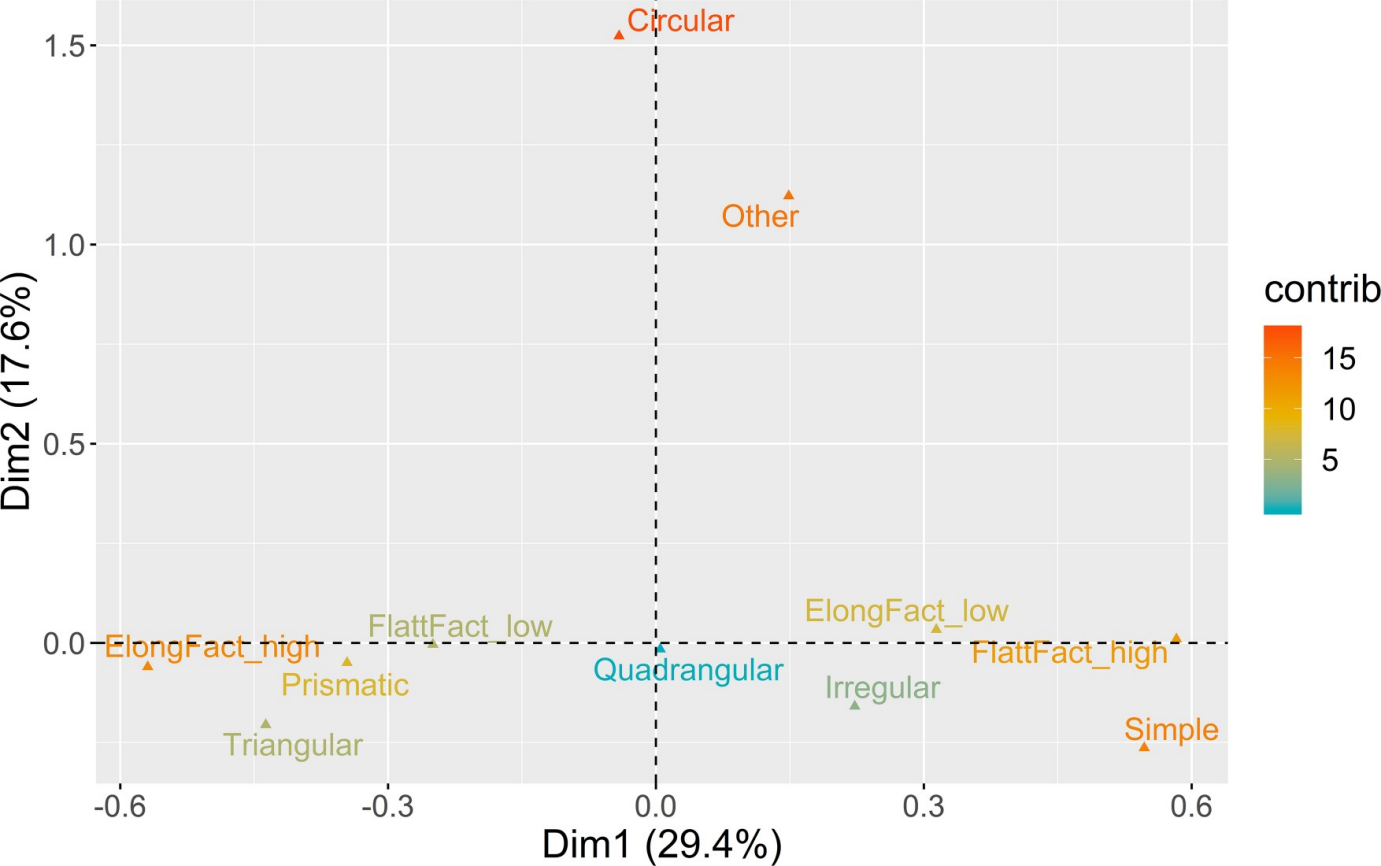
Multiple correspondence analysis plot (first two dimensions) for core morphology. The color ramp represents the contribution (in %) of the variable categories to the definition of the dimensions.

**Fig 14:**
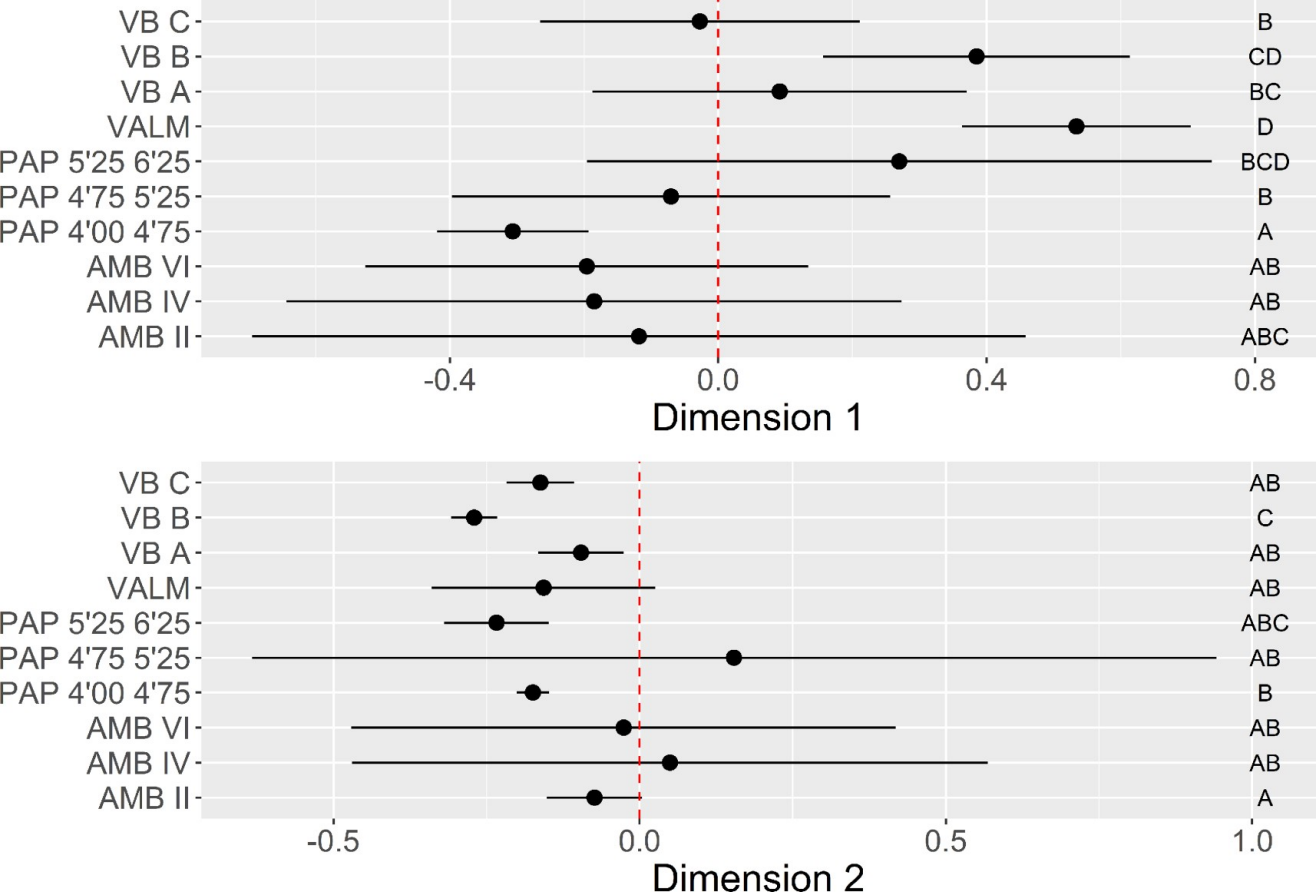
Core morphology multi-comparison bootstrap ANOVA. Error bars represent 95% confidence interval from the mean. Contexts sharing the same set of letters do not present statistically significant difference (p-value ≥ 0.05).

The first and second dimensions of the MCA explain c. 47% of the overall variability (Fig 13). Positive loadings in Dimension 1 (29.4%) are related to the presence of flat cores, with irregular cross-section morphologies and random organization of removals. Regarding the negative loadings, Dimension 1 is mostly marked by elongated, prismatic cores, most of the times presenting triangular cross-sections. The ANOVA comparison results suggest no statistically significant difference across most of the assemblages, with all sites presenting a high degree of variability, as shown by the large confidence intervals shown in Fig 14. Still, the presence of prismatic cores seems to be under-represented at the two latest VB levels, VALM and the oldest level of PAP.

Although representing 17.6% of the variability, Dimension 2 is much less informative than Dimension 1. The positive loadings in Dimension 2 are associated with the occurrence of circular or other types of core cross-sections. Only the PAP 4’75–5’25 and AMB IV assemblages show a rather weak association with this specific pattern. All the remaining contexts are indifferentiable in this dimension from a statistical point of view.

### Core use

The analysis of core use intended to understand how cores were reduced through different techniques to manage convexities and shape cores. Platform treatment, the number, and relationship between platforms, and the type of blanks extracted were used for this analysis. Categorized versions of core weight and elongation were also included to understand how technological variables covary with changes in these two metrics. It is noteworthy, however, that this approach is less powerful than the core exploitation using blanks presented above since the observed patterns are coming from a single moment of core reduction when this was abandoned for some reason.

**Fig 15:**
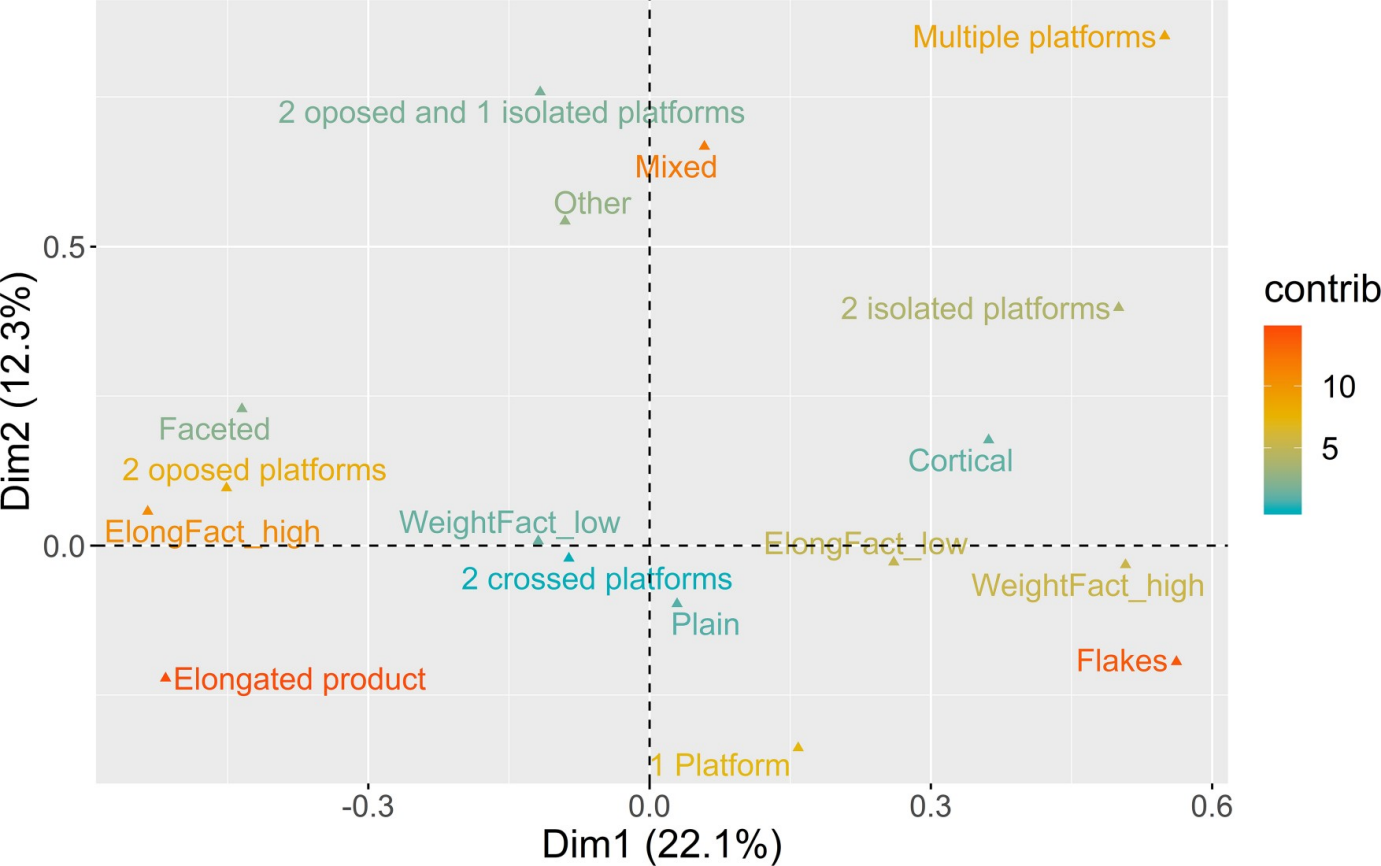
Multiple correspondence analysis plot (first two dimensions) for core use. The color ramp represents the contribution (in %) of the variable categories to the definition of the dimensions.

The total variability calculated by the two first dimensions of the MCA is around 34% (Fig 15).

**Fig 16:**
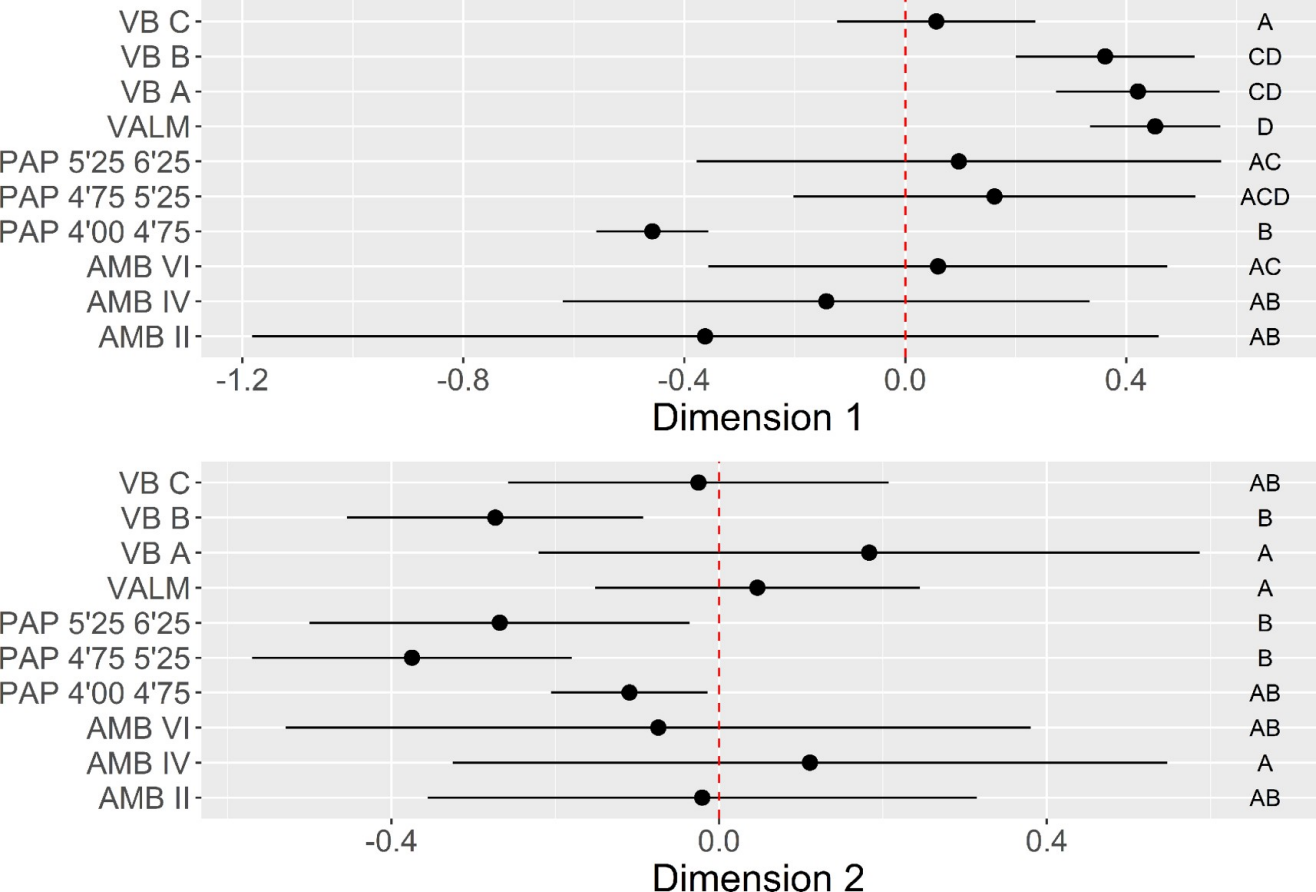
Core use multi-comparison bootstrap ANOVA. Error bars represent 95% confidence interval from the mean. Contexts sharing the same set of letters do not present statistically significant difference (p-value ≥ 0.05).

Dimension 1 (22.1%) is marked by a significant dichotomy between, on the one hand (positive loadings), heavy cores used for the production of flakes, using 2 isolated or multiple platforms, and, on the other hand (negative loadings), highly elongated cores used to extract elongated blanks, using faceted platforms and opposed bidirectional strategies. The plot of trimmed mean values and confidence intervals for each context in Dimension 1 (Fig 16) reveals that the averages of the two latest contexts of AMB and PAP are the ones contributing the most to the negative loadings. On the contrary, VB, VALM and the oldest assemblages of PAP and AMB seem to have reduced frequencies of the component associated with highly elongated blanks and prepared platforms. These results confirm the patterns observed for the platform maintenance and core exploitation using blanks domains presented above, with a well-demarcated chronological division for the latest LGM occupations at PAP and AMB.

In the case of Dimension 2 (12.3%), as in the core morphology analysis, only the positive loadings of the MCA calculation seem to make a relevant contribution to the analysis. These are mostly marked by the occurrence of cores with more than two platforms used for the extraction of mixed products, with no significant correlation showing up between these cores and values for weight and elongation. As expected, the distribution of contexts across this dimension does not seem to reveal any statistically significant differences, suggesting that this technological component occurs equally represented across all sites.

## Discussion and conclusion

**Fig 17:**
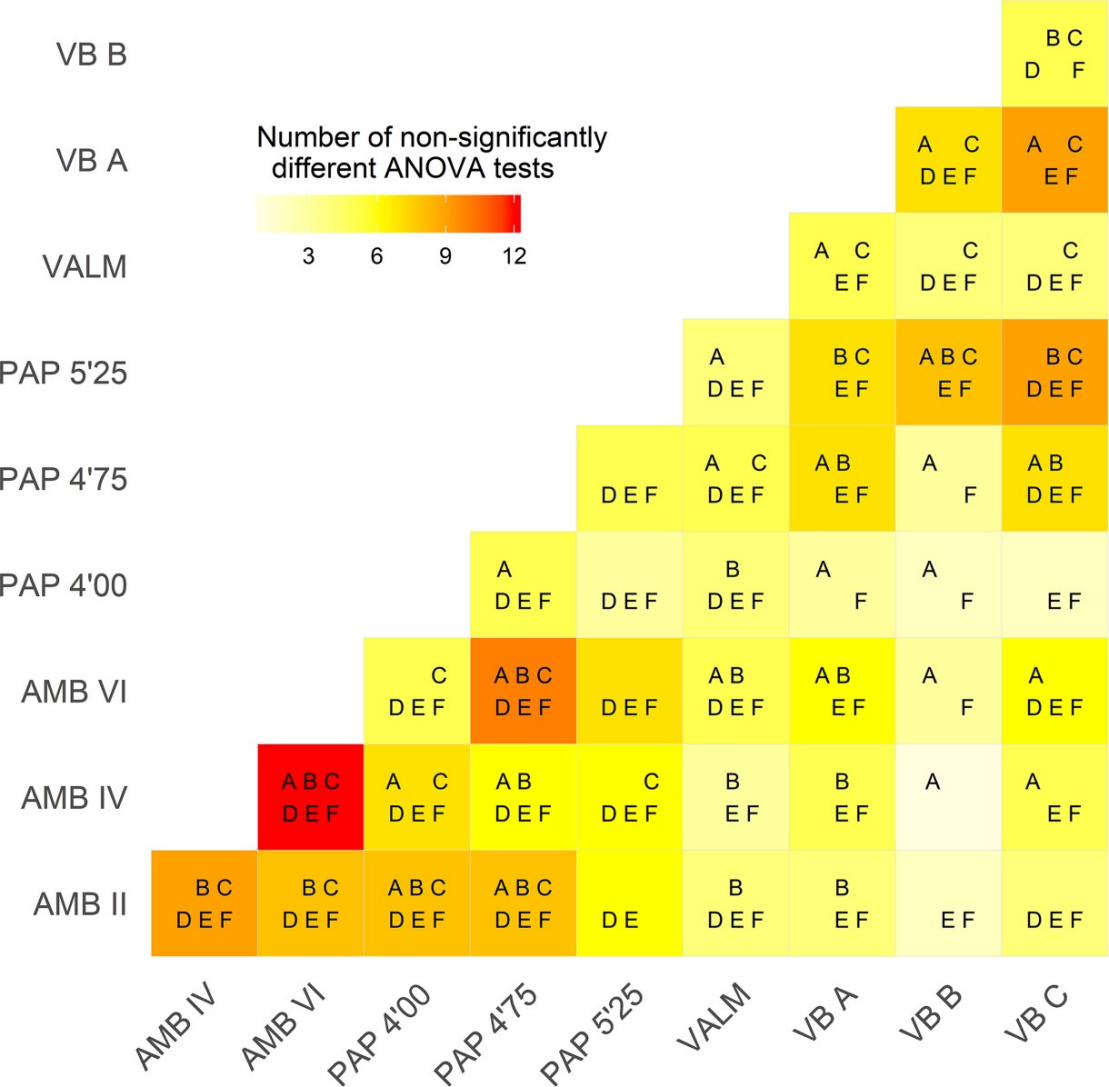
Heat map summarizing the number of non-significantly different bootstrapped ANOVA tests between each pair of assemblages. Letters inside each square indicate the heuristic domains responsible for the similarities between assemblages: A—Platform Maintenance; B—Direction of Core Exploitation; C—Dorsal Surface Convexity; D—Elongated blanks techno-morphology; E—Core Morphology; F—Core Use. For more information on each of the contexts please refer to Table 2.

The analyses presented above aimed to provide a first evaluation of lithic technological variability during the LGM in southern Iberia. Specifically, this study was directed to assess the levels of similarity and difference within and between sites, to better understand the organization of technology in a geographically well-defined cultural entity, in which long-distance contacts and social networks seem to have played a structuring role. The significant variability displayed by the various tests is notable, and particularly evident in the small amount of variation explained by the different MCAs. Nevertheless, preliminary conclusions can be drawn using some of the detected patterns across both time and space. Most of these patterns are better seen in Fig 17, which compiles the overall results of the application of bootstraped ANOVA tests to the mean loadings of each context within the MCAs shown above.

In the first instance, chronological change in the technological options of lithic production is attested by significant dissimilarities between some contexts within multi-layered sites. This pattern is mostly visible in the particularities of platform maintenance and core exploitation domains. However, this seems to have only occurred at AMB and PAP. At AMB, two general technological phases can be distinguished, with results differentiating between the oldest contexts (AMB VI and AMB IV) and the more recent level (AMB II). At PAP, the separation also occurs in the transition between the two oldest levels (PAP 5’25–6’25 and PAP 4’75–5’25) and the most recent one (PAP 4’00–4’75). These disruptions are, at both sites, coincident with the transition from the traditionally-defined Upper Solutrean to Solutreo-Gravettian (or Evolved Upper Solutrean) phases, which is thought to have occurred, at these sites, at around c. 20.5 ka cal BP [43,53,115]. Thus, previous differences detected between those phases, based on typological grounds and pointing to a decline in the use of flat-retouched points and the increasing presence of backed shouldered points [23,116], seem to be corroborated by significant changes in the technological substrate. The most important changes regarding this transition comprise an increasing use of reduction sequences oriented to the production of highly elongated blanks, using opposed bidirectional methods on prismatically-organized cores. The absence of this tendency at VB [27,45] might result from a lack of effective occupation at the site during the later parts of the LGM [45,47], but also by technological drift occuring during this period across Iberia, as suggested by the fact that a Solutreo-Gravettian phase was, so far, not clearly recognized in the Atlantic facade of Iberia.

The distribution of non-significantly different statistical results in Fig 17 reveals also that the degree of similarity between the earliest occupations of the spanish sites and VB is rather high. Particularly, lower blank elongation and simple unidirectional reduction strategies seem to explain the proximity between all levels of VB and the two oldest contexts of PAP and AMB (similar in between c. 75% and c. 60% of the tests, respectively).

According to the traditional chronological scheme of the Solutrean, differences in the extent of social networks in southern and western Iberia occur between the early and later phases of the technocomplex. Zilhão [21], for example, suggests a correlation between global environmental constraints and point types distributions, with epochs of regional convergence broadly coinciding with periods of colder climate (i.e., the Middle Solutrean), and epochs of regional divergence broadly coinciding with periods of more temperate climate (i.e., Upper Solutrean). This relationship can be only explained by fluctuations in size, extension and shape of social networks, with more rigorous environments motivating the existence of long-distance circulation of individuals, objects and ideas, and warmer climates being responsible for decreasing the frequency and intensity of long-distance contacts and favouring cultural drift [25]. Although the results of this study seem to agree with these ideas, they also become problematic when looking at the chronologies and types of lithic projectiles associated with each context. In fact, under the traditional scheme, all three levels of VB are considered Upper Solutrean but appear here correlated with contexts classified as Middle Solutrean (PAP 5’25–6’25 and AMB VI) [43]. Thus, technological convergence cannot be explained by the traditional phased model based on points typology, but only by the association between the two assemblages in terms of absolute chronology [45]. This, in turn, may have implications for the functional organization of settlement within the Mediterranean region that require further dedicated studies to be ascertained conclusively. What seems relevant is that these results open the possibility for the existence of technological convergence across southern Iberia, with contact occuring in a way that enabled sharing of the complete technological solutions. Further evidence for these contacts is provided by the similarities present in other aspects of the archaeological record, such as the style of the animal representations in an engraved slab from VB C and the Middle Solutrean portable art elements in PAP [39,40].

Another relevant, general, trend is the progressive reduction of platform facetting over time at AMB and PAP, even though, with exception of VALM, assemblages present reduced frequencies of platform preparation. Still, although bifacial thinning flakes have been removed from the analysis, the lower percentages of faceted platforms in latest assemblages at AMB and PAP is probably also indicative of a gradual reduction in the importance of bifacial debitage over time [94,117], since the recurrent use of small-scale flaking of striking surfaces to support “on-the-edge” marginal percussion) is typically associated with bifacial knapping strategies [118]. On the other hand, platform preparation through facetting has been shown to relate with a purposeful alteration of exterior platform angles in other type of reduction schemes, to control the size and shape of the extracted blanks. One cannot exclude thus that, in some of the studied assemblages, platform facetting was used to control blanks’ morphometrics. At VALM, the largest frequency of platform facetting preferentially associated with the production of elongated blanks seems to corroborate this interpretation. Thus, the lower frequencies of platform facetting in the remaining assemblages on the one hand, and their significantly high presence at other Solutrean sites in the portuguese Estremadura [24] on the other hand, seems to indicate the existence of particular technological solutions in central Portugal that might imply lower degrees of technological knowledge sharing with the southernmost regions of Iberia. Although some aspects of the singularity of VALM might originate from a biased collection of the materials during the excavation, already mentioned by Zilhão [24], or even from possible differences arising from the single-horizon open-air nature of VALM *contra* the palimpsest-derived assemblages of the remaining sites, the technological dissimilarities detected in this study seem to corroborate the isolation of central Portugal as an independent techno-cultural unit.

The Atlantic vs Mediterranean division is also noteworthy in the overall higher degree of similarity between the two spanish sites, than between those sites and the portuguese assemblages. In the context of the cultural transmission assumptions used in this study, it is tempting to argue that this pattern might be related with a distance factor, in which geographically closer assemblages tend to present a higher degree of technological similarity. A distance decay factor, in which the similarity of cultural traits is expected to decline over distance and that some degree of spatial autocorrelation should exist, is, in fact, one of the major assumptions in archaeological cultural transmission processes literature [80,82,119,120]. Accepting this interpretation would suggest the existence of marked technological differences between what is traditionally defined as the Mediterranean and the Portuguese Solutrean facies, somehow confirming the typologically-based interpretations.

Finally, it is also noteworthy that, despite the similarities in some technological aspects, all VB contexts tend to appear isolated from the remaining contexts used in this study. In previous studies, technological idiosyncrasies of VB have been noted and are not exclusive for LGM occupations [121]. Possible explanations are related with the low knapping quality of chert, featuring small nodules and volumes highly fractured by tectonics [122], available within the VB raw materials acquisition territory. This most likely led hunter-gatherers to develop specific technological strategies fitted to the characteristics of the raw materials [123]. To some degree, VB technological options are stable across the site’s sequence, and independent of changes introduced in tools shape at the beginning of each Upper Paleolithic techno-complex [124]. In the particular case of the Solutrean and the hypotheses raised by this study, technological practices transmission might have occurred in contexts of a high degree of social intimacy, but regional ecological constraints did not allow the generalization of basic technological patterns.

As in any other geographies and timeframes, technological variation over the LGM in southern Iberia was the result of a complex relationship among patterns of inheritance, interaction, and regional adaptation. Population interaction across larger territories was certainly a key factor of the adaptive response to the impact of climate and landscape change and seem to have functioned through sharing behaviors of stylistic/typological concepts, but also of technological solutions. Concept sharing could have occurred across regions in different acculturation environments, depending on the geographical distance between communities and preferential cultural ties, but also on the impact that local resources could have had in the technological decisions. After all, humans, as other biota, interact with the environment at distinct scales and create self-reinforcing adaptive patterns [124]. The multiple but distinct scales of self-organization during the LGM in southern Iberia, and the distribution of behaviors within and across scales might have been one of the most important generators of resilience for those communities [125].

Only further studies using more data from the regions addressed in this study, and other territories located between them, such asthe Alentejo, southwestern Spain, or Central Iberia [126], will allow the ascertainment of the extension of the several scales of influence and contacts.

## Supporting information

S1 Table Blanks attribute frequency. (PDF)

S2 Table Elongated blanks attribute frequency. (PDF)

S3 Table Cores attribute frequency. (PDF)
