# Supplementary material for: Territoriality and the organization of technology during the Last Glacial Maximum in southwestern Europe
Source: PLoS One. 2019 Dec 11;14(12):e0225828. doi: 10.1371/journal.pone.0225828 (PMC6905521; doi:10.1371/journal.pone.0225828)
Supplement: S1 Table — (PDF) [file pone.0225828.s001.pdf]

Table S1 - Blanks attribute frequency

	AMB II	AMB IV	AMB VI	PAP 4'00-4'75	PAP 4'75-5'25	PAP 5'25-6'25	VALM	VB A	VB B	VB C	Total
Platform type, n (%)											
Cortical	60 (11.3)	62 (11.7)	52 (12.9)	240 (9.2)	114 (17.5)	168 (19.4)	108 (17.7)	97 (20.6)	355 (21.2)	154 (18.6)	1410 (15.4)
Crushed	131 (24.7)	92 (17.4)	59 (14.6)	404 (15.5)	84 (12.9)	112 (12.9)	84 (13.7)	39 (8.3)	147 (8.8)	69 (8.3)	1221 (13.3)
Faceted	33 (6.2)	32 (6.1)	34 (8.4)	82 (3.2)	34 (5.2)	60 (6.9)	133 (21.8)	16 (3.4)	48 (2.9)	26 (3.1)	498 (5.4)
Plain	234 (44.2)	279 (52.8)	194 (48.1)	1474 (56.6)	314 (48.2)	394 (45.4)	200 (32.7)	221 (47.0)	817 (48.8)	416 (50.3)	4543 (49.6)
Other	72 (13.6)	63 (11.9)	64 (15.9)	402 (15.4)	106 (16.3)	134 (15.4)	86 (14.1)	97 (20.6)	307 (18.3)	162 (19.6)	1493 (16.3)
Cross section, n (%)											
Irregular	43 (8.1)	45 (8.5)	37 (9.2)	166 (6.4)	48 (7.4)	103 (11.9)	83 (13.6)	101 (21.5)	296 (17.7)	133 (16.1)	1055 (11.5)
Lenticular	24 (4.5)	48 (9.1)	23 (5.7)	117 (4.5)	70 (10.7)	94 (10.8)	32 (5.2)	70 (14.9)	247 (14.8)	82 (9.9)	807 (8.8)
Other	9 (1.7)	4 (0.8)	7 (1.7)	53 (2.0)	6 (0.9)	4 (0.5)	9 (1.5)	11 (2.3)	34 (2.0)	8 (1.0)	145 (1.6)
Trapezoidal	120 (22.6)	111 (21.0)	93 (23.1)	730 (28.1)	123 (18.9)	129 (14.9)	165 (27.0)	54 (11.5)	162 (9.7)	125 (15.1)	1812 (19.8)
Triangular	334 (63.0)	320 (60.6)	243 (60.3)	1536 (59.0)	405 (62.1)	538 (62.0)	322 (52.7)	234 (49.8)	935 (55.9)	479 (57.9)	5346 (58.3)
Profile, n (%)											
Curved	193 (36.4)	173 (32.8)	124 (30.8)	936 (36.0)	206 (31.6)	179 (20.6)	122 (20.0)	103 (21.9)	321 (19.2)	160 (19.3)	2517 (27.5)
Irregular	35 (6.6)	37 (7.0)	28 (6.9)	131 (5.0)	27 (4.1)	51 (5.9)	52 (8.5)	39 (8.3)	171 (10.2)	74 (8.9)	645 (7.0)
Straight	230 (43.4)	260 (49.2)	226 (56.1)	1267 (48.7)	361 (55.4)	572 (65.9)	355 (58.1)	286 (60.9)	1044 (62.4)	518 (62.6)	5119 (55.9)
Twisted	72 (13.6)	58 (11.0)	25 (6.2)	268 (10.3)	58 (8.9)	66 (7.6)	82 (13.4)	42 (8.9)	138 (8.2)	75 (9.1)	884 (9.6)
Dorsal pattern, n (%)											
Bidirectional	114 (21.5)	88 (16.7)	64 (15.9)	799 (30.7)	135 (20.7)	121 (13.9)	189 (30.9)	57 (12.1)	135 (8.1)	82 (9.9)	1784 (19.5)
Unidentifiable	76 (14.3)	77 (14.6)	53 (13.2)	364 (14.0)	123 (18.9)	173 (19.9)	98 (16.0)	100 (21.3)	451 (26.9)	185 (22.4)	1700 (18.5)
Unidirectional	324 (61.1)	340 (64.4)	264 (65.5)	1342 (51.6)	371 (56.9)	518 (59.7)	281 (46.0)	287 (61.1)	1039 (62.1)	529 (64.0)	5295 (57.8)
Other	16 (3.0)	23 (4.4)	22 (5.5)	97 (3.7)	23 (3.5)	56 (6.5)	43 (7.0)	26 (5.5)	49 (2.9)	31 (3.7)	386 (4.2)
Edge shape, n (%)											
Biconvex	19 (3.6)	28 (5.3)	31 (7.7)	154 (5.9)	64 (9.8)	64 (7.4)	44 (7.2)	17 (3.6)	82 (4.9)	41 (5.0)	544 (5.9)
Convergent	41 (7.7)	45 (8.5)	36 (8.9)	175 (6.7)	48 (7.4)	50 (5.8)	41 (6.7)	75 (16.0)	219 (13.1)	73 (8.8)	803 (8.8)
Divergent	91 (17.2)	89 (16.9)	82 (20.3)	331 (12.7)	148 (22.7)	178 (20.5)	143 (23.4)	102 (21.7)	333 (19.9)	206 (24.9)	1703 (18.6)
Irregular	139 (26.2)	160 (30.3)	110 (27.3)	824 (31.7)	149 (22.9)	285 (32.8)	239 (39.1)	135 (28.7)	516 (30.8)	255 (30.8)	2812 (30.7)
Parallel	174 (32.8)	141 (26.7)	106 (26.3)	836 (32.1)	177 (27.1)	206 (23.7)	131 (21.4)	112 (23.8)	429 (25.6)	208 (25.2)	2520 (27.5)
Other	66 (12.5)	65 (12.3)	38 (9.4)	282 (10.8)	66 (10.1)	85 (9.8)	13 (2.1)	29 (6.2)	95 (5.7)	44 (5.3)	783 (8.5)
Cortex %, n (%)											
0%	354 (66.8)	342 (64.8)	243 (60.3)	1705 (65.5)	364 (55.8)	418 (48.2)	381 (62.4)	289 (61.5)	912 (54.5)	486 (58.8)	5494 (59.9)
1-25%	65 (12.3)	76 (14.4)	53 (13.2)	394 (15.1)	99 (15.2)	128 (14.7)	85 (13.9)	52 (11.1)	157 (9.4)	88 (10.6)	1197 (13.1)
26-75%	84 (15.8)	70 (13.3)	72 (17.9)	337 (13.0)	106 (16.3)	232 (26.7)	104 (17.0)	61 (13.0)	288 (17.2)	138 (16.7)	1492 (16.3)
76-100%	27 (5.1)	40 (7.6)	35 (8.7)	166 (6.4)	83 (12.7)	90 (10.4)	41 (6.7)	68 (14.5)	317 (18.9)	115 (13.9)	982 (10.7)
Termination, n (%)											
Feathered	463 (87.4)	468 (88.6)	357 (88.6)	2321 (89.2)	574 (88.0)	786 (90.6)	521 (85.3)	361 (76.8)	1385 (82.7)	698 (84.4)	7934 (86.6)
Pointed	41 (7.7)	26 (4.9)	20 (5.0)	220 (8.5)	44 (6.7)	40 (4.6)	54 (8.8)	76 (16.2)	211 (12.6)	81 (9.8)	813 (8.9)
Other	26 (4.9)	34 (6.4)	26 (6.5)	61 (2.3)	34 (5.2)	42 (4.8)	36 (5.9)	33 (7.0)	78 (4.7)	48 (5.8)	418 (4.6)
Elongation, M (SD)	2.34 (1.16)	1.83 (0.86)	1.80 (0.88)	2.42 (1.12)	1.73 (0.94)	1.46 (0.67)	1.68 (0.87)	1.54 (0.67)	1.46 (0.71)	1.46 (0.72)	1.86 (1.00)
Flattening, M (SD)	4.40 (2.14)	4.52 (2.19)	4.04 (1.80)	4.02 (1.61)	4.48 (2.22)	4.27 (1.98)	4.29 (1.78)	3.92 (2.21)	3.95 (2.32)	3.98 (2.13)	4.12 (2.01)
