# Supplementary material for: Territoriality and the organization of technology during the Last Glacial Maximum in southwestern Europe
Source: PLoS One. 2019 Dec 11;14(12):e0225828. doi: 10.1371/journal.pone.0225828 (PMC6905521; doi:10.1371/journal.pone.0225828)
Supplement: S2 Table — (PDF) [file pone.0225828.s002.pdf]

Table S2 - Elongated blanks attribute frequency

	AMB II	AMB IV	AMB VI	PAP 4'00-4'75	PAP 4'75-5'25	PAP 5'25-6'25	VALM	VB A	VB B	VB C	Total
Platform type, n (%)											
Cortical	18 (6.1)	12 (6.7)	4 (3.4)	89 (5.7)	20 (11.8)	10 (8.3)	9 (5.1)	10 (10.8)	33 (11.4)	14 (9.8)	219 (6.9)
Crushed	79 (26.9)	44 (24.4)	28 (23.7)	273 (17.4)	23 (13.6)	24 (19.8)	32 (18.2)	13 (14.0)	44 (15.2)	18 (12.6)	578 (18.3)
Dihedral	5 (1.7)	5 (2.8)	4 (3.4)	31 (2.0)	3 (1.8)	0 (0.0)	3 (1.7)	6 (6.5)	12 (4.2)	10 (7.0)	79 (2.5)
Faceted	18 (6.1)	18 (10.0)	6 (5.1)	33 (2.1)	10 (5.9)	8 (6.6)	65 (36.9)	3 (3.2)	9 (3.1)	3 (2.1)	173 (5.5)
Other	32 (10.9)	15 (8.3)	15 (12.7)	212 (13.5)	27 (16.0)	10 (8.3)	22 (12.5)	21 (22.6)	63 (21.8)	28 (19.6)	445 (14.1)
Plain	142 (48.3)	86 (47.8)	61 (51.7)	933 (59.4)	86 (50.9)	69 (57.0)	45 (25.6)	40 (43.0)	128 (44.3)	70 (49.0)	1660 (52.6)
Cross section, n (%)											
Trapezoidal	78 (26.5)	41 (22.8)	45 (38.1)	588 (37.4)	65 (38.5)	32 (26.4)	80 (45.5)	19 (20.4)	48 (16.6)	33 (23.1)	1029 (32.6)
Triangular	207 (70.4)	135 (75.0)	67 (56.8)	940 (59.8)	100 (59.2)	87 (71.9)	90 (51.1)	68 (73.1)	214 (74.0)	103 (72.0)	2011 (63.8)
Other	9 (3.1)	4 (2.2)	6 (5.1)	43 (2.7)	4 (2.4)	2 (1.7)	6 (3.4)	6 (6.5)	27 (9.3)	7 (4.9)	114 (3.6)
Profile, n (%)											
Curved	118 (40.1)	65 (36.1)	45 (38.1)	665 (42.3)	79 (46.7)	57 (47.1)	57 (32.4)	20 (21.5)	68 (23.5)	38 (26.6)	1212 (38.4)
Irregular	17 (5.8)	6 (3.3)	4 (3.4)	72 (4.6)	5 (3.0)	4 (3.3)	6 (3.4)	4 (4.3)	9 (3.1)	4 (2.8)	131 (4.2)
Straight	114 (38.8)	82 (45.6)	54 (45.8)	627 (39.9)	62 (36.7)	41 (33.9)	71 (40.3)	55 (59.1)	171 (59.2)	78 (54.5)	1355 (43.0)
Twisted	45 (15.3)	27 (15.0)	15 (12.7)	207 (13.2)	23 (13.6)	19 (15.7)	42 (23.9)	14 (15.1)	41 (14.2)	23 (16.1)	456 (14.5)
Dorsal pattern, n (%)											
Bidirectional	93 (31.6)	60 (33.3)	33 (28.0)	681 (43.3)	87 (51.5)	38 (31.4)	96 (54.5)	10 (10.8)	51 (17.6)	28 (19.6)	1177 (37.3)
Other	27 (9.2)	7 (3.9)	5 (4.2)	137 (8.7)	13 (7.7)	15 (12.4)	17 (9.7)	9 (9.7)	34 (11.8)	10 (7.0)	274 (8.7)
Unidirectional	174 (59.2)	113 (62.8)	80 (67.8)	753 (47.9)	69 (40.8)	68 (56.2)	63 (35.8)	74 (79.6)	204 (70.6)	105 (73.4)	1703 (54.0)
Edge shape, n (%)											
Biconvex	7 (2.4)	9 (5.0)	2 (1.7)	81 (5.2)	14 (8.3)	8 (6.6)	9 (5.1)	2 (2.2)	21 (7.3)	7 (4.9)	160 (5.1)
Convergent	30 (10.2)	24 (13.3)	22 (18.6)	127 (8.1)	20 (11.8)	8 (6.6)	20 (11.4)	31 (33.3)	67 (23.2)	19 (13.3)	368 (11.7)
Divergent	19 (6.5)	19 (10.6)	9 (7.6)	115 (7.3)	13 (7.7)	6 (5.0)	16 (9.1)	11 (11.8)	30 (10.4)	12 (8.4)	250 (7.9)
Irregular	66 (22.4)	47 (26.1)	29 (24.6)	466 (29.7)	37 (21.9)	40 (33.1)	71 (40.3)	15 (16.1)	51 (17.6)	29 (20.3)	851 (27.0)
Parallel	123 (41.8)	66 (36.7)	48 (40.7)	643 (40.9)	75 (44.4)	55 (45.5)	60 (34.1)	34 (36.6)	119 (41.2)	74 (51.7)	1297 (41.1)
Other	49 (16.7)	15 (8.3)	8 (6.8)	139 (8.8)	10 (5.9)	4 (3.3)	0 (0.0)	0 (0.0)	1 (0.3)	2 (1.4)	228 (7.2)
Cortex %, n (%)											
0%	246 (83.7)	145 (80.6)	89 (75.4)	1270 (80.8)	125 (74.0)	76 (62.8)	141 (80.1)	57 (61.3)	199 (68.9)	104 (72.7)	2452 (77.7)
1-25%	31 (10.5)	19 (10.6)	16 (13.6)	212 (13.5)	31 (18.3)	25 (20.7)	16 (9.1)	19 (20.4)	39 (13.5)	20 (14.0)	428 (13.6)
26-75%	14 (4.8)	14 (7.8)	12 (10.2)	77 (4.9)	10 (5.9)	16 (13.2)	14 (8.0)	15 (16.1)	38 (13.1)	12 (8.4)	222 (7.0)
76-100%	3 (1.0)	2 (1.1)	1 (0.8)	12 (0.8)	3 (1.8)	4 (3.3)	5 (2.8)	2 (2.2)	13 (4.5)	7 (4.9)	52 (1.6)
Termination, n (%)											
Feathered	240 (81.6)	151 (83.9)	93 (78.8)	1325 (84.3)	138 (81.7)	103 (85.1)	135 (76.7)	41 (44.1)	170 (58.8)	100 (69.9)	2496 (79.1)
Pointed	39 (13.3)	21 (11.7)	15 (12.7)	205 (13.0)	26 (15.4)	17 (14.0)	34 (19.3)	46 (49.5)	107 (37.0)	33 (23.1)	543 (17.2)
Other	15 (5.1)	8 (4.4)	10 (8.5)	41 (2.6)	5 (3.0)	1 (0.8)	7 (4.0)	6 (6.5)	12 (4.2)	10 (7.0)	115 (3.6)
Elongation, M (SD)	3.11 (0.95)	2.75 (0.69)	2.88 (0.74)	3.13 (0.81)	3.00 (0.83)	2.71 (0.67)	2.79 (0.68)	2.45 (0.50)	2.55 (0.59)	2.59 (0.69)	2.96 (0.81)
Flattening, M (SD)	3.66 (1.18)	3.76 (1.18)	3.60 (1.14)	3.60 (1.10)	3.88 (2.29)	3.42 (1.15)	3.95 (1.30)	2.86 (1.31)	3.15 (1.43)	3.19 (1.28)	3.56 (1.28)
