# Supplementary material for: Territoriality and the organization of technology during the Last Glacial Maximum in southwestern Europe
Source: PLoS One. 2019 Dec 11;14(12):e0225828. doi: 10.1371/journal.pone.0225828 (PMC6905521; doi:10.1371/journal.pone.0225828)
Supplement: S3 Table — (PDF) [file pone.0225828.s003.pdf]

Table S3 - Cores attribute frequency

	AMB II	AMB IV	AMB VI	PAP 4'00-4'75	PAP 4'75-5'25	PAP 5'25-6'25	VALM	VB A	VB B	VB C	Total
Core type, n (%)											
Prismatic	7 (77.8)	10 (71.4)	8 (61.5)	84 (74.3)	6 (54.5)	3 (20.0)	18 (32.7)	12 (60.0)	11 (23.9)	18 (58.1)	177 (54.1)
Simple	1 (11.1)	2 (14.3)	2 (15.4)	24 (21.2)	4 (36.4)	10 (66.7)	26 (47.3)	5 (25.0)	33 (71.7)	9 (29.0)	116 (35.5)
Other	1 (11.1)	2 (14.3)	3 (23.1)	5 (4.4)	1 (9.1)	2 (13.3)	11 (20.0)	3 (15.0)	2 (4.3)	4 (12.9)	34 (10.4)
Platforms, n (%)											
1 Platform	2 (22.2)	5 (35.7)	8 (61.5)	40 (35.4)	8 (72.7)	7 (46.7)	20 (36.4)	7 (35.0)	32 (69.6)	20 (64.5)	149 (45.6)
2 crossed platforms	0 (0.0)	0 (0.0)	0 (0.0)	0 (0.0)	0 (0.0)	1 (6.7)	1 (1.8)	0 (0.0)	0 (0.0)	1 (3.2)	3 (0.9)
2 isolated platforms	0 (0.0)	1 (7.1)	0 (0.0)	3 (2.7)	1 (9.1)	1 (6.7)	9 (16.4)	5 (25.0)	8 (17.4)	1 (3.2)	29 (8.9)
2 oposed and 1 isolated platforms	0 (0.0)	1 (7.1)	0 (0.0)	3 (2.7)	0 (0.0)	0 (0.0)	3 (5.5)	1 (5.0)	1 (2.2)	0 (0.0)	9 (2.8)
2 oposed platforms	5 (55.6)	6 (42.9)	2 (15.4)	64 (56.6)	1 (9.1)	5 (33.3)	14 (25.5)	4 (20.0)	5 (10.9)	6 (19.4)	112 (34.3)
Multiple platforms	2 (22.2)	1 (7.1)	3 (23.1)	3 (2.7)	1 (9.1)	1 (6.7)	8 (14.5)	3 (15.0)	0 (0.0)	3 (9.7)	25 (7.6)
Core section, n (%)											
Circular	1 (11.1)	2 (14.3)	1 (7.7)	9 (8.0)	3 (27.3)	0 (0.0)	3 (5.5)	3 (15.0)	0 (0.0)	1 (3.2)	23 (7.0)
Irregular	3 (33.3)	5 (35.7)	4 (30.8)	43 (38.1)	5 (45.5)	2 (13.3)	24 (43.6)	5 (25.0)	13 (28.3)	16 (51.6)	120 (36.7)
Quadrangular	5 (55.6)	4 (28.6)	6 (46.2)	39 (34.5)	2 (18.2)	10 (66.7)	22 (40.0)	10 (50.0)	21 (45.7)	11 (35.5)	130 (39.8)
Triangular	0 (0.0)	3 (21.4)	2 (15.4)	22 (19.5)	1 (9.1)	3 (20.0)	6 (10.9)	2 (10.0)	12 (26.1)	3 (9.7)	54 (16.5)
Blank type, n (%)											
Elongated product	7 (77.8)	7 (50.0)	5 (38.5)	83 (73.5)	3 (27.3)	6 (40.0)	6 (10.9)	1 (5.0)	9 (19.6)	7 (22.6)	134 (41.0)
Flakes	1 (11.1)	2 (14.3)	6 (46.2)	10 (8.8)	7 (63.6)	9 (60.0)	32 (58.2)	11 (55.0)	27 (58.7)	10 (32.3)	115 (35.2)
Mixed	1 (11.1)	5 (35.7)	2 (15.4)	20 (17.7)	1 (9.1)	0 (0.0)	17 (30.9)	8 (40.0)	10 (21.7)	14 (45.2)	78 (23.9)
Cortex %, n (%)											
0%	1 (11.1)	5 (35.7)	3 (23.1)	17 (15.0)	7 (63.6)	2 (13.3)	7 (12.7)	7 (35.0)	10 (21.7)	7 (22.6)	66 (20.2)
1-25%	4 (44.4)	4 (28.6)	5 (38.5)	41 (36.3)	1 (9.1)	3 (20.0)	19 (34.5)	7 (35.0)	19 (41.3)	12 (38.7)	115 (35.2)
26-75%	4 (44.4)	5 (35.7)	5 (38.5)	49 (43.4)	3 (27.3)	10 (66.7)	28 (50.9)	6 (30.0)	16 (34.8)	9 (29.0)	135 (41.3)
76-100%	0 (0.0)	0 (0.0)	0 (0.0)	6 (5.3)	0 (0.0)	0 (0.0)	1 (1.8)	0 (0.0)	1 (2.2)	3 (9.7)	11 (3.4)
Weight, M (SD)	40.3 (34.0)	37.6 (21.4)	31.7 (19.2)	14.3 (10.0)	11.5 (6.2)	16.2 (4.7)	30.2 (21.2)	25.1 (29.3)	19.5 (14.8)	22.3 (12.4)	21.5 (18.1)
Elongation, M (SD)	1.20 (0.30)	1.30 (0.52)	1.12 (0.43)	1.35 (0.37)	1.01 (0.33)	1.24 (0.44)	0.95 (0.37)	1.01 (0.37)	1.03 (0.39)	1.20 (0.44)	1.17 (0.42)
Flattening, M (SD)	1.54 (0.36)	1.43 (0.56)	1.34 (0.30)	1.41 (0.45)	1.27 (0.48)	1.51 (0.68)	2.04 (0.78)	1.74 (0.71)	1.77 (0.72)	1.49 (0.52)	1.59 (0.64)
